# Latitudinal Clines in Climate and Sleep Patterns Shape Disease Outcomes in *Drosophila melanogaster* Infected by *Metarhizium anisopliae*

**DOI:** 10.1002/ece3.71047

**Published:** 2025-02-28

**Authors:** Mintong Nan, Jonathan B. Wang, Michail Siokis, Raymond J. St. Leger

**Affiliations:** ^1^ Department of Entomology University of Maryland College Park Maryland USA

**Keywords:** biomes and disease resistance, disease resistance and global *Drosophila* populations, *Drosophila* sickness sleep and disease resistance, geography and sleep patterns, joint clinal evolution of sleep and disease resistance, *Metarhizium anisopliae* fungal infection, sex and mating status modulate infection

## Abstract

Major latitudinal clines have been observed in *Drosophila melanogaster* , a human commensal that originated in tropical Africa and has subsequently dispersed globally to colonize temperate habitats. However, despite the crucial role pathogens play in species distribution, our understanding of how geographical factors influence disease susceptibility remains limited. This investigation explored the effects of latitudinal clines and biomes on disease resistance using the common fly pathogen *Metarhizium anisopliae* and 43 global *Drosophila melanogaster* populations. The findings revealed correlations between disease resistance and latitudinal gradients of sleep duration, temperature, and humidity. Although enhanced defenses may be driven by fungal diversity at tropical latitudes, the most disease‐resistant tropical males also showed the highest susceptibility to desiccation. This suggests potential trade‐offs between abiotic stress resistance, necessary for survival in temperate habitats, and disease resistance. Furthermore, the study uncovered interactions between sex, mating status, sleep, and abiotic stresses, affecting disease resistance. Notably, longer‐sleeping males and virgin flies survived infections longer, with additional daytime sleep post‐infection being protective, particularly in the most resistant fly lines. These observations support the hypothesis that sleep and disease defense are intertwined traits linked to organismal fitness and subject to joint clinal evolution.

## Graphical abstract

We examined how environmental and genetic factors influence the ability of *Drosophila melanogaster* populations to defend themselves against a common fly pathogen. Tropical flies, particularly males and virgins (of either sex) slept more and were more resistant to disease than seasonal populations, indicating that geographical location, sex, mating status, and sleep interact to influence disease resistance. This research addresses themes of species adaptations in diverse ecological settings, the evolution of host‐pathogen interactions, and how closely related individuals exposed to the same infection can have different outcomes.

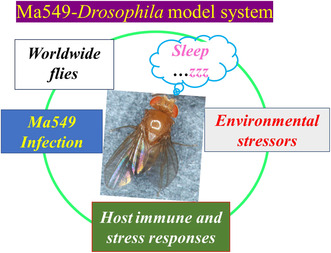

## 1 Introduction

Understanding how organisms adapt to their environment, especially which biological processes are most relevant, remains an unresolved question in biology (Mateo et al. 2018) and one that has become increasingly critical due to the escalating threats to biodiversity from human activities. The fruit fly *Drosophila melanogaster* , a key evolutionary model, presents exceptional opportunities to investigate historical processes experienced by human commensals (Arguello et al. 2019). This species, which originated in sub‐Saharan Africa and initially specialized in marula fruit ( *Sclerocarya birrea* ), first associated with people when marula was stored by cave‐dwellers (Mansourian et al. 2018). Notably, flies maintained under laboratory conditions since 1916 still demonstrate a preference for marula over other fruits (Mansourian et al. 2018). The expansion of *D. melanogaster* 's range to the Middle East occurred around 13,000 years ago, followed by its spread to Europe and Asia about 1800 years ago, coinciding with citrus and grape cultivation (Sprengelmeyer et al. 2019). Subsequent colonization events, driven by human dispersal, have spread fly populations globally, including North America and Australia about 200 years ago (David and Capy 1988; Haudry et al. 2020). The colonization of diverse natural habitats, environmental clines, and geographic variables across continents indicates *D. melanogaster* 's capacity to rapidly adapt to local conditions (Mateo et al. 2018; Blanford et al. 2003). This adaptability establishes *D. melanogaster* as a valuable model for studying evolutionary processes of range expansion and adaptation (Arguello et al. 2019) offering insights into evolutionary responses to future environmental challenges. Studies of *D. melanogaster* lines from diverse locations have revealed sleep differences (Brown et al. 2018) linked to metabolic and foraging needs (Keene and Duboue 2018) and associated with latitudinal clines and elevated temperatures (Brown et al. 2018). While other clines, such as body size and chill resistance, which vary predictably with abiotic factors, have been extensively studied theoretically and experimentally, comparatively little is understood about clines whose evolution may be influenced by interspecific interactions.

Flies potentially encounter distinct parasitic and pathogenic pressures in different environments, and according to the Red Queen hypothesis, hosts continually evolve to minimize the effects of pathogen adaptation (Dybdahl and Lively 1998; Lively and Dybdahl 2000; Blanford et al. 2003). Disease resistance varies across *Drosophila* species; those with a broad diet (e.g., *D. melanogaster, D. simulans, D. repleta, D. arizonae*) typically being more resistant to *Metarhizium anisopliae* strain Ma549 than dietary specialists (e.g., *D. erecta, D. sechellia*) (O'Malley et al. 2023). This phenomenon may be attributed to the increased exposure of insects with a broad diet to a wider range of pathogens facilitating the development of resistance (Wang et al. 2017; O'Malley et al. 2023). There is also a great deal of intraspecific variation in disease resistance in several species (Wang et al. 2017; O'Malley et al. 2023), although the environmental predictability of this variation remains largely unexplored. Notably, four African *D. melanogaster* lines demonstrated superior resistance to the fungal pathogen *Beauveria bassiana* compared to two non‐African populations (Tinsley et al. 2006).

While the ecological and evolutionary processes underlying disease resistance are understudied, extensive research has been conducted on the molecular mechanisms of host–pathogen interactions using *D. melanogaster* (Westlake et al. 2024; Lemaitre and Hoffmann 2007; Igboin et al. 2012). These studies have revealed significant conservation of pathogenesis and host defense mechanisms between higher host organisms and *Drosophila* (Ugur et al. 2016; Panayidou et al. 2014). Nonetheless, both individual fruit flies and humans exhibit considerable variability in their responses to infectious agents (Råberg et al. 2007; Lu and St Leger 2016), as evidenced by the diverse human responses to COVID‐19. This variability underscores the importance of elucidating the genetic, environmental, and biological factors that contribute to differential responses to disease (Guilger‐Casagrande et al. 2021). A central question in infection biology is why individuals exposed to identical infections experience different outcomes (Duneau et al. 2017), but it is likely attributable to their distinct evolutionary histories.

Fungi cause the majority of insect diseases (Faria and Wraight 2007), infecting their hosts by directly penetrating the cuticle with a combination of cuticle‐degrading enzymes and mechanical pressure (St. Leger 2024; Wang and St. Leger 2006). The entomopathogenic fungus *M. anisopliae* is widespread globally, and strain ARSEF549 (Ma549) is naturally pathogenic to *Drosophila* (Lu et al. 2015). Unlike many other entomopathogens, Ma549 does not produce toxins that suppress innate immune responses (Pal et al. 2007). Previous studies have shown that about 9% of mutant *D. melanogaster* lines exhibit altered disease resistance to Ma549 (Lu et al. 2015) and that *Drosophila* Genetic Reference Panel (DGRP) flies from Raleigh, North Carolina, display significant variation in longevity after Ma549 infection (Wang et al. 2017). A GWAS analysis of DGRP flies revealed a complex network of immune and physiological genes, particularly those related to sleep, involved in *D. melanogaster* interactions with Ma549 (Wang et al. 2017). However, DGRP data are limited to genetic variation from a single location (North‐Eastern USA), while a major portion of the genetic diversity of the species resides elsewhere, notably in its ancestral home Africa (Hutter et al. 2007). Thus, DGRP data alone are inadequate for comprehensively studying global pathogen susceptibility.

The intricate relationship between organisms and their habitats is fundamental to ecological and environmental studies. The complexity of this relationship is exemplified by diverse biomes, each with unique environmental conditions (Kreft and Jetz 2007). Aseasonal climates are found in biomes such as tropical and subtropical moist broadleaf forests (TSMF), tropical and subtropical grasslands, Savannas and shrublands (TSGSS) (Dinerstein et al. 2017), and tropical and subtropical dry broadleaf forests (TSDF). Conversely, biomes like temperate broadleaf and mixed forests (TBMF), Mediterranean forests, woodlands, and scrub (MFWS), and Desert Xeric Shrublands (DXS) exhibit greater seasonal variability. The unique climatic, topographic, and ecological characteristics of each biome significantly influence resident organisms (Kreft and Jetz 2007; Vasar et al. 2022), including *D. melanogaster* populations and fungal species, with tropical regions harboring a greater diversity and abundance of pathogens (Vasar et al. 2022; Tedersoo et al. 2014; Větrovský et al. 2019).

This investigation employed Ma549 and 43 global *D. melanogaster* populations to examine the effects of environmental factors, genetic diversity, and sleep behavior on disease resistance. The research emphasizes temperature and humidity due to their substantial influence on both fungal infection prevalence and insect resistance (Athanassiou et al. 2017). Climate change‐induced increases in temperature, alterations in precipitation patterns, and extreme events pose a threat to the survival of numerous insects and their pathogens (St. Leger 2021). Consequently, understanding adaptive responses to these stressors is crucial for predicting climate change impacts (Kellermann et al. 2018). We checked the relationship between abiotic and biotic stress using flies from opposite ends of the resistance and biome spectrum. Specifically, Monkey Hills (MH), St. Kitts, characterized by a humid and stable TSMF biome (Stancioff et al. 2018), and Ica, Peru (IP), a hyper‐arid landscape with significant climatic variability (Salem 1989) were selected for particular analysis.

The findings demonstrate significant differences in Ma549 resistance among *Drosophila* populations from diverse global sites. This variation correlates with geographic origins and ecoregional conditions, indicating persistent macro‐physiological relationships between disease resistance and other traits. Sexual dimorphism influences host‐pathogen interactions irrespective of geographic origin, with infection having a more pronounced effect on male sleep patterns. Additionally, sexual activity impacts disease resistance and sleep patterns, highlighting the complex interplay of genetic, environmental, and behavioral factors in shaping host responses to pathogens.

## 2 Materials and Methods

### 2.1 Fly Stocks, Geographic and Climate Data, and Biomes

Twenty‐two fly populations were a gift from Dr. Alex Keene's lab (Texas A&M University) (Brown et al. 2018) and an additional 21 fly populations [Ghana (Mataheko or Dansoman, Accra), Mauritius (Le Reduit), Malawi (Lujeri), Madagascar (Tananarive), France (Montpellier), and the United Kingdom (Sussex)] were acquired from the *Drosophila* Species Stock Center (Cornell University, Ithaca, NY) with all stock numbers given in Table 1. The lines were obtained when the center was in Illinois (before the move to Cornell). Judging by the dates given by the stock center, the collection labs seem to have passed the lines to them soon after capture. *Drosophila* were cultured on cornmeal‐molasses media supplemented with yeast, agar, Tegosept, and propionic acid at 25°C and ∼70% humidity under a 12‐h light:dark cycle. Virgin flies of each genotype were collected under light CO_2_ anesthesia within 8 h of eclosion and housed in separate vials for 2–3 days to mature.

**TABLE 1:** The localities, stock numbers, Ma549 LT_50_s, collection years, and geographic variables (including biomes) of 43 *Drosophila melanogaster* populations.

Fly lines	Stock	Female			Male			Collection date	Lat.	Lon.	Alt.	Biomes
		LT_50_	±SEM	*N*	LT_50_	±SEM	*N*					
American Samoa	134	5.28	0.12	326	5.48	0.04	359	2009	−14.30	−170.70	157	TSMF
Bogota, Colombia	59	5.44	0.10	345	5.27	0.10	390	1962	4.71	−74.07	2258	TSMF
Cebu, Philippines	129	4.73	0.04	433	4.49	0.03	394	2008	10.32	123.89	35	TSMF
Le Reduit, Mauritius	53	5.44	0.09	365	6.24	0.09	376	2006	−20.23	57.49	282	TSMF
Monkey Hill, St. Kitts	35	5.28	0.18	501	8.09	0.13	373	2005	17.32	−62.73	121	TSMF
Ogasawara Islands, Japan	137	5.83	0.09	356	6.03	0.15	322	2009	27.08	142.21	152	TSMF
Tananarive, Madagascar	125	4.53	0.08	357	5.47	0.11	398	1982	−18.87	47.51	1250	TSMF
Bahia, Brazil	15	4.00	0.07	295	4.10	0.03	365	Not Listed	−12.58	−41.70	907	TSDF
Bermuda	58	4.57	0.13	446	4.40	0.08	497	1954	32.31	−64.75	14	TSDF
Chiapas, Mexico	22	4.72	0.05	321	4.94	0.15	280	2002	16.76	−93.13	536	TSDF
Accra, Ghana.163	163	5.84	0.08	396	6.86	0.13	354	2010	5.60	−0.19	54	TSGSS
Accra, Ghana.165	165	5.24	0.05	387	6.29	0.07	395	2010	5.60	−0.19	54	TSGSS
Accra, Ghana.167	167	5.25	0.04	360	6.10	0.06	305	2010	5.60	−0.19	54	TSGSS
Accra, Ghana.169	169	6.25	0.08	404	7.16	0.05	279	2010	5.60	−0.19	54	TSGSS
Accra, Ghana.171	171	5.68	0.06	359	7.22	0.11	312	2010	5.60	−0.19	54	TSGSS
Accra, Ghana.173	173	5.20	0.06	332	6.74	0.10	337	2010	5.60	−0.19	54	TSGSS
Accra, Ghana.175	175	5.34	0.07	281	6.08	0.08	292	2010	5.60	−0.19	54	TSGSS
Accra, Ghana.177	177	5.57	0.05	375	6.18	0.12	350	2010	5.60	−0.19	54	TSGSS
Accra, Ghana.179	179	5.23	0.04	338	6.76	0.17	311	2010	5.60	−0.19	54	TSGSS
Accra, Ghana.181	181	5.59	0.05	350	5.77	0.08	346	2010	5.60	−0.19	54	TSGSS
Kariba Dam, Zimbabwe	64	4.46	0.09	304	5.23	0.07	337	1963	−16.52	28.76	485	TSGSS
Lujeri, Malawi	76	5.11	0.10	369	5.04	0.10	329	2009	−16.04	35.65	717	TSGSS
Queensland, Australia	03	4.55	0.13	327	5.60	0.16	330	Not Listed	−20.92	142.70	185	TSGSS
Ica, Peru	01	3.43	0.08	281	3.96	0.05	344	1956	−14.08	−75.73	403	DXS
Israel	68	3.95	0.09	323	4.35	0.09	336	1954	31.05	34.85	485	DXS
San Luis Potosi, Mexico	43	4.68	0.09	330	5.38	0.20	314	2005	22.16	−100.99	1878	DXS
Athens, Greece	69	5.01	0.09	340	5.99	0.10	310	1965	37.98	23.73	69	MFWS
Cape Town, South Africa	62	5.73	0.17	324	6.93	0.14	327	1954	−33.92	18.42	7	MFWS
Crete, Greece	23	4.30	0.06	422	4.34	0.04	352	2002	35.24	24.81	1667	MFWS
Montpellier, France.139	139	4.76	0.04	375	6.47	0.10	377	2010	43.62	3.86	47	MFWS
Montpellier, France.140	140	4.79	0.04	392	5.08	0.10	382	2010	43.62	3.86	47	MFWS
Montpellier, France.141	141	4.14	0.10	353	4.72	0.13	377	2010	43.62	3.86	47	MFWS
Montpellier, France.142	142	4.28	0.10	337	6.18	0.13	333	2010	43.62	3.86	47	MFWS
Montpellier, France.143	143	4.43	0.08	376	4.70	0.05	365	2010	43.62	3.86	47	MFWS
Montpellier, France.144	144	4.17	0.08	336	4.19	0.05	302	2010	43.62	3.86	47	MFWS
Blacksburg, Virginia	61	4.06	0.04	328	4.85	0.15	346	1954	37.23	−80.41	641	TBMF
Fukushima, Japan	136	3.70	0.08	335	4.90	0.10	290	2009	37.76	140.47	65	TBMF
Plainville, Connecticut	56	4.93	0.10	370	5.72	0.10	437	2007	41.68	−72.86	54	TBMF
Pyrenees, Spain	67	4.59	0.16	282	4.95	0.08	319	1965	42.67	1.00	2311	TBMF
Queensferry, Scotland	130	4.72	0.06	325	5.15	0.08	358	2009	55.99	−3.40	25	TBMF
Southwest Harbor, Maine	132	5.06	0.07	321	5.41	0.13	331	2009	44.28	−68.33	13	TBMF
Sussex, United Kingdom.161	161	3.87	0.05	377	4.30	0.10	364	2011	50.97	−0.47	53	TBMF
Sussex, United Kingdom.162	162	4.45	0.05	433	4.78	0.10	362	2011	50.97	−0.47	53	TBMF

*Note:* Stocks all contain 14021‐0231 prefixes.

Abbreviations: Alt., Altitude; DXS, Deserts and Xeric Shrublands; Lat., Latitude; Lon., Longitude; MFWS, Mediterranean Forests, Woodlands, and Scrub; TBMF, Temperate Broadleaf and Mixed Forests; TSDF, Tropical and Subtropical Dry broadleaf Forests; TSGSS, Tropical and Subtropical Grasslands, Savannas, and Shrublands; TSMF, Tropical and Subtropical Moist broadleaf Forests.

Latitude, longitude, and altitude measurements of each collection site in Table 1 were obtained from Google Earth Pro and converted from degrees, minutes, seconds to decimal degrees. Latitude and altitude measurements were transformed into absolute values, followed by conversion to log_10_ for regression analysis. The 43 fly populations were categorized into six biomes based on the latitude and longitude of each collection site using ArcGIS Pro (2022, Version 3.0.1, Esri Inc., Redlands, CA, USA) and 14 biomes defined in Ecoregions 2017 (Dinerstein et al. 2017). Because latitude, altitude, and precipitation measurements were not normally distributed, we calculated the log_10_ values for the Ma549 LT_50_ regression analyses. The geographic data in Figure 2A were generated using ArcGIS Pro.

Monthly average, high (daytime), and low (nighttime) temperature datasets of land and ocean in a 1° × 1° latitude‐longitude grid covering the Earth's surface from 1850 to the present were obtained from the Berkeley Earth website (http://berkeleyearth.org/data/) and verified using the visualization tool L3HARRIS IDL software (https://www.l3harrisgeospatial.com/Software‐Technology/IDL). Temperature data based on the collection year of all 28 collection sites were in a gridded NetCDF format and extracted with a self‐coded program script (Appendix S1: Table A2B), as provided in Appendix S1: Table A2A. The annual average, high, and low temperatures were computed by summing the respective monthly averages, highs, and lows and dividing each sum by 12.

The monthly precipitation data within a 0.5° grid for each collection site and collection year in Appendix S1: Table A2A were extracted from a large database of up to 10 different weather stations at the World Meteorological Organization (Schneider et al. 2020). The annual mean precipitation was determined by averaging the monthly data across all 12 months. The month with the highest precipitation value among the 12 months was identified as the wettest month.

Data on 52,584 hourly temperatures and relative humidity from the years 2005–2010 for all 28 collection sites defined by latitude and longitude were obtained from the Prediction of Worldwide Energy Resource (POWER) Project, Langley Research Center, National Aeronautics and Space Administration. To cover all 43 fly populations, we used NASA POWER data to analyze hourly temperature and humidity from 2005 to 2010 at 28 collection sites, aligning with the collection dates of most fly lines (Appendix S1: Table A3). These data were utilized to analyze the effects of environmental factors on fly longevity. The total hours during which temperatures fell within specific ranges and relative humidity within 60%–100% from 2005 to 2010 across all 28 collection sites were calculated using 52,584 hourly temperature and humidity data points. The total hours were then divided by 6 years to calculate the annual hourly duration (hours/year) for 43 fly lines, which were used in simple linear regression analyses.

### 2.2 Fungal Strains and Longevity Bioassays

*Metarhizium anisopliae* 549 (Ma549) was acquired from the ARS Collection of Entomopathogenic Fungal Cultures (ARSEF) (Ithaca, NY). Ma549 cultures were thawed from −80°C stock vials and grown for 10–14 days at 27°C on potato‐dextrose‐agar media plates.

Bioassays using *M. anisopliae* 549 transformed to express green fluorescent protein (Ma549‐GFP) were performed according to a previous report (Lu et al. 2015) on 43 worldwide fly populations. Briefly, fly populations were infected at the same time of day, and survival was monitored after topical inoculation in groups of five replicates (20–30 flies each) per sex per line. Each line was screened three times. To prepare inoculum, conidia were suspended in sterile distilled water, vortexed for 2 min and filtered through Miracloth (22–25 μm) (Andwin Scientific). Flies (2 to 4 days old) were vortexed with spore suspensions (20 mL, 2.5 × 10^4^ conidia/mL) for 30 s (~inoculum load 200 spores per fly), collected by filtering through Miracloth and transferred into vials containing fly stock food without Tegosept and propionic acid. Flies were cultured at 27°C and ~85% relative humidity. Male and female flies were housed in separate food vials to examine sexual dimorphism and avoid an interaction between the effects of sex and age in response to infection. Less than 10% of flies vortexed with water alone (mock‐infected) or conidial suspensions died within 1 day, and there were no significant differences between lines; therefore, flies succumbing within 1 day post‐infection were deleted from the infection data. The flies were flipped into fresh food vials daily. The number of dead flies was recorded twice daily for 10–14 days. Host survival differences were measured as LT_50_ values (median lethal time in days at which 50% of the flies died) calculated using R 4.4.2 (R Core Team 2024). This method was highly reproducible, with a mean LT_50_ value for control *Drs*‐GFP flies of 4.682 ± 0.029 (*N* = 126). The inoculum load of spores per male and female *Drosophila* was approximately 200 colony‐forming units (Lu et al. 2015). To quantify micro‐environmental plasticity in mean survival time (MST) as described in a previous report (Morgante et al. 2015), we used either the untransformed or transformed within‐line standard deviation (*σ*_*E*_) and coefficient of environmental variation (*CV*_*E*_) (Appendix S1: Table A1B), depending on normality tests. *σ*_*E*_ has the advantage of being measured in days post‐infection, the same scale as the trait mean, while *CV*_*E*_ is calculated as the standard deviation divided by the mean and multiplied by 100.

### 2.3 Desiccation Assays

Ten age‐matched (3 to 6 days old) flies were sorted by sex using light CO_2_ anesthesia and placed into a 25 × 95 mm tube, covered at the top with gauze, and joined with parafilm to another 25 × 95 mm tube containing 10 g of silica gel desiccant beads (Wisedry B01MPYB16J) at the bottom to achieve a desiccated relative humidity of 5%–10%. Two replicates per sex per line were used for each experimental trial. Control chambers contained 5 mL of a supersaturated NaCl solution to maintain a stable relative humidity of approximately 70%. The flies were monitored at 1‐h intervals for 20 h for death, as indicated by failure to right themselves or to move their legs when their vials were tapped or inverted (Gibbs et al. 1997).

### 2.4 Fungal Colonization of Hosts Measured by Colony Forming Units (CFUs)

A time‐course bioassay of fungal growth in the hemolymph of mated and virgin MH, CG, and IP females and males was conducted using previously described protocols (Lu et al. 2015). Fifteen flies per sex, mating status, and line at each time point across three experiments were individually homogenized with 45 μL of 0.1% Tween 80. For the resistant MH line, the entire 45 μL homogenate was spread onto *Metarhizium* selective medium (Rose Bengal Agar plates supplemented with ox bile, CTAB, oxytetracycline, streptomycin, penicillin, chloramphenicol, and cycloheximide; Hu and St Leger 2002). For susceptible lines, 5 μL of a 10‐fold dilution of the homogenate was spread onto the plates. Colony forming units (CFUs) were counted using the ImageJ Cell Counter after up to 7 days of incubation at 25°C.

### 2.5 Sleep Assays and Data Analysis

The *Drosophila* Activity Monitor (DAM2) from Trikinetics, Waltham, MA, was used to monitor locomotor activity and analyze sleep patterns in MH (resistant), IP (susceptible), and CG (average longevity) flies. Flies were individually housed in 5 × 65 mm monitor tubes containing 5% sucrose and 2% agar after CO_2_ anesthesia, with any initial mortalities replaced within 12 h to minimize acclimation effects. Monitoring occurred over 7 days under a 12‐h light/dark (LD) cycle (lights on at 9:00 AM EDT, lights off at 9:00 PM EDT). Sleep data, defined as periods of 5 min or more of inactivity (Shaw et al. 2000), were processed using DAMFileScan113 software (Trikinetics) and analyzed using custom software (Insomniac3, RP Metrix, Skillman, NJ; gift of Dr. Julie Williams, University of Pennsylvania). The DAM2 output was converted to total daily, nighttime, and daytime sleep duration, represented by the number of minutes when a fly was asleep in 24 or 12 h. Sleep patterns were examined from 12 to 60 h post‐infection with Ma549, spanning two light–dark cycles. This period precedes the onset of most visible symptoms, such as reduced feeding and reproductive behavior, and the upregulation of the immune gene *Drosomycin* in response to Ma549 infection (Lu et al. 2015; Wang et al. 2023). The sleep profiles of mated CG and IP flies were compared with those of MH flies under normal conditions, and infected flies were compared with uninfected peers to assess the impact of infection within each line. Sex‐specific differences in sleep and disease outcomes were also investigated along with the influence of mating status on disease resistance and sleep patterns. Sleep differences were quantified using the net percentage differences between the experimental groups (Appendix S1: Table A5).

### 2.6 Statistical Analyses on Interactions Among Clinal Cues, Geographic Variables, Longevity, and Sleep Parameters

We conducted simple linear regression analyses between LT_50_ values and environmental cues or geographic variables (altitude and latitude) at collection sites for 43 global (subdivided into 23 aseasonal and 20 seasonal) populations. We also correlated LT_50_ values with sleep parameters data for 22 female fly populations (Brown et al. 2018). Pearson's correlation analyses were performed in GraphPad Prism 10 (GraphPad Software, Boston, MA, USA).

To analyze fly survival data after Ma549 infection, we used a Cox mixed‐effects model (coxme): Surv(Time, event) ~ Sex + Mating Status + Sex:Mating Status + (1|Line), where Time represents survival time, event is survival status, Sex and Mating Status are fixed effects, and their interaction term (Sex:Mating Status) tests for combined effects. Line accounts for geographical variability as a random effect. Pairwise comparisons of hazard ratios were performed using the emmeans package in R with Tukey's correction (*p* < 0.05): emmeans (coxme_model, pairwise ~ Sex + Mating Status + Sex:Mating Status, type = “response”). Survival curves are plotted as Kaplan–Meier plots, and the model provided hazard ratios with confidence intervals to evaluate the impact of sex, mating status, and genetic factors on fly survival.

We evaluated data normality using the D'Agostino‐Pearson and Shapiro–Wilk normality tests, known for their robustness across various statistical distributions (Yap and Sim 2011). For data sets passing at least one normality test and showing statistically similar standard deviations (SDs), we employed the unpaired Student's *t*‐test for two group comparisons and one‐way ANOVA (Tukey's multiple comparisons) for three or more groups. If variances significantly differed (*p* < 0.05), we used Welch's correction (Welch's *t*‐test) for two groups and the one‐way Welch's ANOVA (Dunnett's multiple comparisons) for multiple groups. For data sets failing normality tests, we used the Mann–Whitney *U* test for two groups and the Kruskal–Wallis test with Dunn's post hoc test for three or more groups. Reported values included *p*‐values, median ± IQR (interquartile range: the difference between the 75th and 25th percentile) from the Kruskal–Wallis test with Dunn's post hoc multiple comparisons test, and mean ± SEM from ordinary ANOVA or Welch's test. Statistical significance was set at *p* < 0.05.

JMP Pro, version 17.1 (SAS Institute Inc., Cary, NC, USA) was utilized for the forward stepwise regression analysis on the impact of geography and biome on resistance levels (Table 3), principal component loadings of environmental variables and LT_50_ for male and female flies (Table 4), as well as ANOVA involving four factors and generating interaction profiles (Appendices S10 and S11).

## 3 Results

### 3.1 The Longevity of Infected Fruit Flies Varies by Collection Site and Sex

We assessed the ability of 43 age‐matched fly lines/populations from diverse geographic regions to survive infection by Ma549. Infection was performed via the natural route of applying spores to the body surface. Median lethal times (LT_50_) were monitored using five replicates (~35 flies each) per sex per fly line, and the experiments were conducted at least twice. Lines with an unusually large disparity between males and females were screened three times to validate differences. A total of 14,957 male flies and 15,287 female flies were assayed, and the collection locations for the fly populations are listed in Table 1 and Appendix S1: Table A1.

The 43 fly populations were sorted into six biomes based on the latitude and longitude of each collection site using ArcGIS Pro, and the 14 biomes defined in Ecoregions 2017 (Dinerstein et al. 2017) as follows: TSMF (Tropical and Subtropical Moist broadleaf Forests), TSDF (Tropical and Subtropical Dry broadleaf Forests), TSGSS (Tropical and Subtropical Grasslands, Savannas, and Shrublands), DXS (Deserts and Xeric Shrublands), MFWS (Mediterranean Forests, Woodlands, and Scrub), and TBMF (Temperate Broadleaf and Mixed Forests).

**FIGURE 1:**
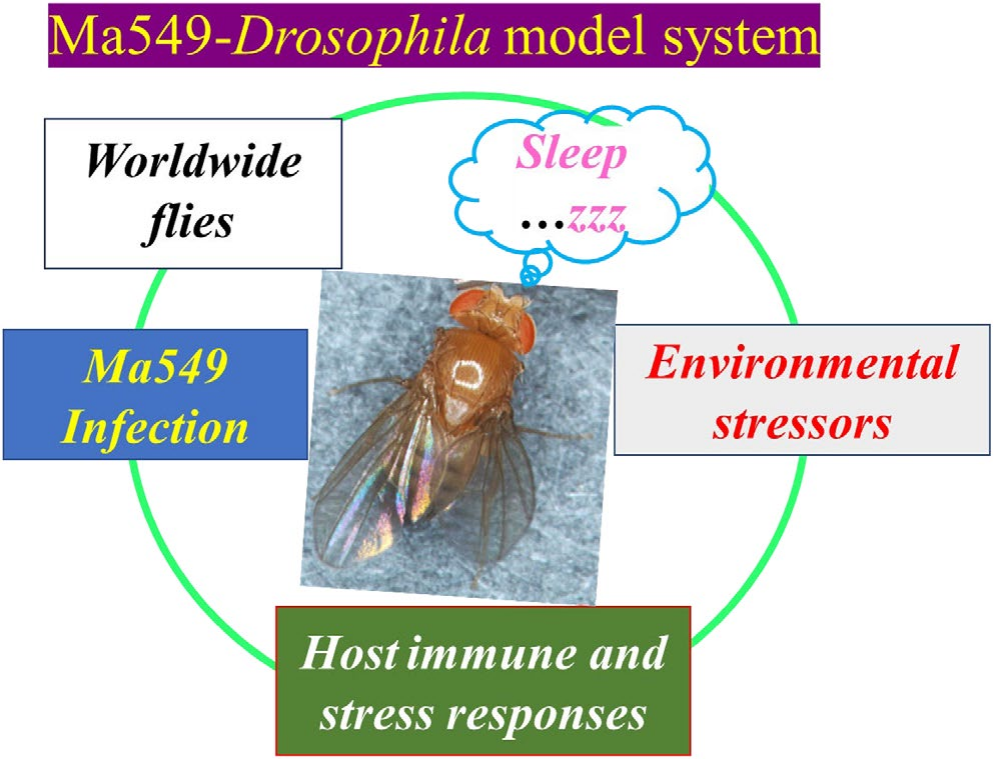
Overview of the Ma549‐*Drosophila* model system. The figure highlights the components of the Ma549‐*Drosophila* system used to study host immune and stress responses. This model incorporates global fly populations (“Worldwide flies”), Ma549 infection, environmental stressors, and sleep behavior. Together, these factors provide a comprehensive framework for investigating the interplay between infection, stress, and immune responses in *Drosophila*.

**FIGURE 2:**
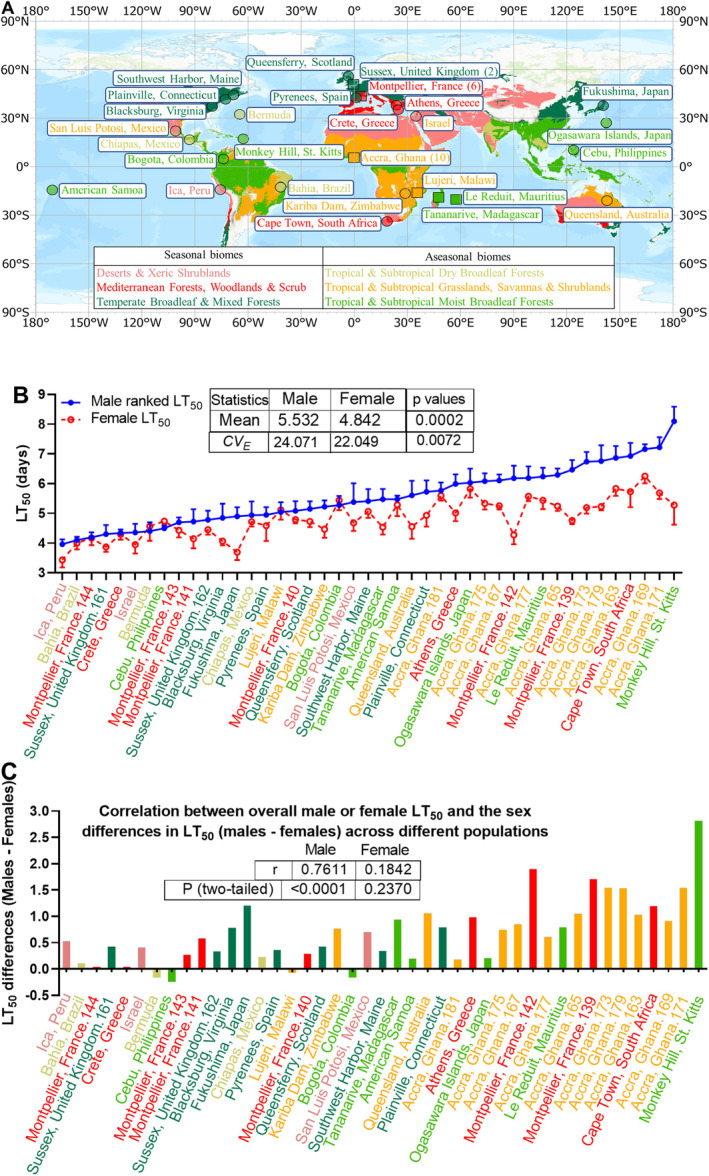
(A) Collection locations and biomes of *Drosophila melanogaster* populations. The geographic and biomes map (Dinerstein et al. 2017) generated by ArcGIS Pro displays the collection locations and biomes of 43 fly populations. The biomes are color coded for this and subsequent figures: Bright green for tropical and subtropical moist broadleaf forests (TSMF), light olive for tropical and subtropical dry broadleaf forests (TSDF), gold for tropical & subtropical grasslands, savannas and shrublands (TSGSS), pink for deserts and xeric shrublands (DXS), red for mediterranean forests, woodlands and scrub (MFWS), and forest green for temperate broadleaf and mixed forests (TBMF). The numbers in parentheses represent the number of fly lines collected at each site. The 22 circles mark the collection sites of previously deployed fly stocks (Brown et al. 2018) (gifted by Dr. Alex Keene), while six squares denote collection sites of 21 fly stocks from the *Drosophila* Species Stock Center. (B) The graph shows the mean LT_50_ values for females (red circles) and males (blue dots) across all 43 global fly lines, ranked by males from least to most resistant to Ma549 infection. Welch's *t* test and unpaired *t*‐test were used to obtain *p*‐values for mean and coefficient of variation (*CV*_*E*_), respectively. (C) Sex differences in LT_50_ sorted in the same order as panel B to facilitate trend comparison. The graph illustrates sex‐based disparities in Ma549 LT_50_ values, calculated by subtracting female LT_50_ values from male LT_50_ values for each of the 43 lines, averaged from 10 to 15 replicates (each containing an average of 35 flies), per sex per line in 2–3 experiments. Positive values indicate greater resistance in males, while negative values indicate greater resistance in females.

To examine interactions among fungal infection, sleep, and host defense in populations of worldwide *Drosophila melanogaster* (Figure 1), we also subdivided the 43 lines into four distinct geographical regions based on their collection sites: African (15), European/Middle East (13), Asian/Pacific (4), and American (11). The African lines were from Ghana (10), Madagascar (1), Malawi (1), Mauritius (1), South Africa (1), and Zimbabwe (1), spanning the TSMF, TSGSS, and MFWS biomes. The European/Middle East lines were from England (2), France (6), Greece (2), Israel (1), Scotland (1), and Spain (1), covering the DXS, MFWS, and TBMF biomes. The Asian/Pacific lines were from Australia (1), Japan (2), and the Philippines (1), representing the TSMF, TSGSS, and TBMF biomes. The American lines were from Bermuda (1), Brazil (1), Colombia (1), Mexico (2), Peru (1), and various locations in the United States (5), encompassing the TSMF, TSDF, DXS, and TBMF biomes (Table 1, Figure 2A).

Sexual dimorphism was investigated by evaluating resistance to Ma549 separately in males and females. Two‐way ANOVA revealed significant interaction effects between line/genetic factor and sex (*F*_(42,800)_ = 17.49, *p* < 0.0001), accounting for 9.87% of the total variation. Genetic factors exerted the greatest influence (*F*_(42,800)_ = 117.3, *p* < 0.0001), explaining 66.17% of the total variation, while sex contributed 12.81% (*F*_(1,800)_ = 953.5, *p* < 0.0001; Appendix S1: Tables A1A, A8A, and Appendix S3A). This indicates that despite males generally exhibiting longer lifespans, the extent of differences between sexes varies significantly across strains. Strong correlations between mean survival time (MST) and LT_50_ values were observed for both males (*r* = 0.9963, *p* < 0.0001) and females (*r* = 0.9992, *p* < 0.0001; Appendix S3B), confirming both metrics as reliable measures of disease resistance. We mostly used LT_50_ values to compare resistance across lines and MST values for analyses related to plasticity.

Kaplan–Meier survival plots confirmed a sex‐based disparity in fly longevity, with males outliving females in 39 of 43 lines (Appendix S1: Table A1A, Appendix S3A and Figure 2B,C). LT_50_ values for both sexes across diverse geographical regions exhibited normal distributions (Appendix S3C). The global average LT_50_ for males and females was 5.53 and 4.84 days, respectively, indicating males were 1.14‐fold more resistant on average (*t* = 3.871, *p* = 0.0002, Figure 2B). The range between the most resistant and susceptible males (females) spanned 4.13 (2.82) days, from 3.96 (3.43) to 8.09 (6.25) days (Figure 2B,C and Appendix S3C, Table 1), demonstrating a broader spectrum of LT_50_ values in male flies. Nevertheless, we observed strong cross‐sex LT_50_ correlations (*r* = 0.7778, *p* < 0.0001, Appendix S3D), closely aligning with DGRP lines 0.7374, *p* < 0.0001 (Wang et al. 2017).

The most susceptible line originated from Ica, Peru (DXS biome), with an LT_50_ of 3.96 and 3.43 days for males and females, respectively. The most resistant male flies were obtained from Monkey Hill (MH), St. Kitts (TMSF biome) with an LT_50_ of 8.09 days (Figure 2B, Table 1). Males from the MH populations exhibited a 1.53‐fold increase in resistance compared to MH females, resulting in MH flies having the largest sexual disparity of 2.81 days (Figure 2C, Table 1). Four lines (Cebu/Philippines, Bogota/Colombia, Bermuda, and Lujeri/Malawi) with intermediate LT_50_ values showed longer female lifespans (Figure 2C, Table 1), although these longevity differences were only significant in the Cebu/Philippines line (*t* = 3.68, *p* = 0.0003, Appendix S2). Correlation analysis (Figure 2B,C) revealed a strong positive correlation between male LT₅₀ and sex‐based survival disparities (*r* = 0.7611, *p* < 0.0001), indicating that higher male LT₅₀ values correspond to larger survival disparities favoring males over females. In contrast, female LT₅₀ values showed no significant correlation with sex differences (*r* = 0.1842, *p* = 0.2370). These results suggest that sex differences in survival are more pronounced in populations with higher male resistance, while they diminish in highly susceptible populations where both sexes die quickly.

Ectothermic organisms commonly exhibit phenotypic plasticity, allowing a single genotype to produce different phenotypes to adapt to environmental stressors, such as temperature (Morgante et al. 2015). This plasticity is typically measured through variability among individuals (Morgante et al. 2015). While the global lines are highly inbred, they are not necessarily clones; therefore, following common practice, we estimated phenotypic plasticity using within‐line variability expressed as a coefficient of environmental variation (*CV*_*E*_), which removes the association between mean and variance (David et al. 2005). The within‐line standard deviation (*σ*_*E*_) has been used to measure variability among individuals in clonal lines of the DGRP (Morgante et al. 2015), and we utilized *σ*_*E*_ to demonstrate that plasticity in disease resistance is highly heritable (Wang et al. 2017). In this study, we found that *σ*_*E*_ positively (*p* < 0.0001) correlates with *CV*_*E*_ (males *r* = 0.7605; females *r* = 0.7157) and MST values (males *r* = 0.8300; females *r* = 0.5652), indicating that the most resistant lines also have the greatest variance (Appendix S4A,B).

However, *CV*_*E*_ is not significantly correlated with MST values in males (*r* = 0.2786, *p* = 0.0705) or females (*r* = − 0.1634, *p* = 0.2952) (Appendix S4C), a pattern generally observed for mean and *CV*_*E*_ plasticity estimates in *D. melanogaster* (David et al. 2005). This suggests that disease resistance plasticity is largely independent of dimension, consistent with different genes controlling plasticity and MST values as previously found in the DGRP (Wang et al. 2017). The *CV*_*E*_ ranged from 17.68 to 32.56 in males and 14.35 to 29.36 in females. Levene's test was used to compare sex‐based variations in disease resistance (Appendix S2). Of the 43 strains, 27 had higher *CV*_*E*_ in males than females, with 23 showing significant differences per Levene's test. Variation between sexes across the 43 lines was assessed by comparing *CV*_*E*_ using a paired *t*‐test, revealing significantly larger male variance across lines (*t* = 2.755, *p* = 0.0072, Appendix S2), confirming sex‐ and line‐specific differences in variance. MH males exhibited the third highest plasticity and IP males the second lowest *CV*_*E*_ (Appendix S1: Table A1B, *p* ≤ 0.0024, Appendix S4D,E), while for *σ*_*E*_, MH males were the most plastic and IP males the least (Appendix S1: Table A1B, *p* < 0.0001, Appendix S4F,G).

### 3.2 Fly Line and Sex Matter More Than Collection Date

As the 43 fly lines had been inbred in laboratories for 13–70 years (Table 1), we investigated whether domestication contributed to their variable longevity following infection. Excluding two lines with unspecified collection dates (Bahia/Brazil and Queensland/Australia), we analyzed the remaining 41 lines.

To assess the effect of fly line, sex, and collection date on fly longevity (LT_50_) following Ma549 infection, we used a linear mixed model formulated as: $Y_{{\mathrm{LT}}_{50}}$ *= μ + S + C + S × C + L + ε*, where $Y_{{\mathrm{LT}}_{50}}$ is the response variable representing LT50 values, *μ* is the overall mean, *S* denotes the fixed effect of sex, *C* represents the fixed effect of collection date, *S* × *C* indicates the interaction term between sex and collection date, *L* is the random effect of the fly lines, and *ε* is the residual error (Tables 2A and 2B).

**TABLE 2A:** Fixed effect tests on longevity effects of sex and collection date.

Source	Nparm	DF	DFDen	*F* ratio	*p*
Sex	1	1	41.79	20.9029	< 0.0001
Collection date	14	14	27.47	1.2574	0.2935
Sex*Collection date	14	14	28.16	1.3107	0.2618

*Source:* This is a list of the tested factors. Nparm: number of parameters associated with the effect. DF: degrees of freedom for each source of variation. DFDen: denominator degrees of freedom associated with the error term or residuals. *F* ratio: the mean square of the factor divided by the mean square of the error. A *p* value less than 0.05 means that the factor has a statistically significant effect.

**TABLE 2B:** REML variance component estimates on longevity effects of fly lines.

Random effect	Variance ratio	Variance component	Standard Error	95% Lower	95% Upper	*p*	% Of total
Fly lines	2.4757	0.4393	0.1457	0.1537	0.7245	0.0026	71.23
Residual		0.1775	0.0483	0.1109	0.3288		28.77
Total		0.6168	0.1457	0.4075	1.0423		100.00

*Note:* REML, restricted maximum likelihood, is a statistical method used to estimate variance components in linear mixed models, providing unbiased estimates by accounting for the loss of degrees of freedom when estimating fixed effects.

The fixed effects model revealed significant main effects of sex (*F*_(1,41.79)_ = 20.90, *p* < 0.0001) but not collection date (*F*_(14,27.47)_ = 1.26, *p* = 0.2935) on LT_50_ values (Table 2A). Additionally, no significant interaction between sex and collection date was observed (*F*_(14,28.16)_ = 1.31, *p* = 0.2618). Therefore, the significant effect of sex on LT_50_ values is independent of the collection date.

### 3.3 Geography and Biomes Impact on Host Susceptibility to Infection

Dividing the 43 global fly populations into four geographic regions (Africa, Europe/Middle East, Asia/Pacific, and America) and six biomes allowed us to determine the relative contributions of geographic location and biome characteristics in disease resistance. Stepwise regression analysis showed significant effects of both factors (*p* < 0.0001, Tables 1 and 3), with biome effects (*η* ^2^ = 0.1291 or 12.91%) approximately doubling those of geography (*η* ^2^ = 0.0624 or 6.24%).

**TABLE 3:** Impact of geography and biome on resistance levels.

Source	Nparm	DF	Sum of squares	*F* ratio	*p*	*η* ^2^ effect size
Geography	3	3	49.79355	28.7166	< 0.0001	0.0624
Biome	5	5	103.07443	35.6666	< 0.0001	0.1291

*Source:* The two tested factors. Nparm: number of parameters associated with the effect. DF: degrees of freedom for each source of variation. Sum of squares: sum of squares (SS) for each source of variation, along with the total from all sources. *F* ratio: the mean square of the factor divided by the mean square of the error. *η* ^2^ (eta squared) is a measure of effect size for analysis of variance (ANOVA) models. A *p* value less than 0.05 means that the factor has a statistically significant effect.

African populations, comprising 35% of the 43 lines, had mean LT_50_ values of 5.98 days (males) and 5.65 days (females), showing 1.12–1.24‐fold greater disease resistance than other regions, including the North Carolina DGRPs (Wang et al. 2017) (*p* ≤ 0.0484, Appendix S5, and Appendix S1: Table A8B). While males consistently outlived females by 1.06 to 1.07‐fold within each region (*p* < 0.0001), African females outlived European/Middle Eastern males by 1.16‐fold (*p* = 0.0021).

### 3.4 Environmental Factors at the Original Collection Sites Influence Host Susceptibility to Infection in the Laboratory

We focused on temperature and humidity, key abiotic factors (Lotterhos et al. 2018) that act as stressors for many organisms, especially ectotherms when outside their preferred ranges (Bogaerts‐Márquez et al. 2021). Latitude strongly correlates with annual temperature minimums and ranges, while altitude moderately correlates with annual relative humidity minimums and ranges (Appendix S6A–F). A linear decrease in relative humidity with increasing altitude (Peixoto and Oort 1996) shows altitude's indirect influence on latitudinal humidity differences.

The clinal patterns in disease resistance for the 43 fly populations are shown in Appendix S7A–G. A pronounced linear decrease in disease resistance (Ma549 LT_50_ values) with increasing latitude is evident in both females (*R* ^2^ = 0.3342, *p* < 0.0001) and males (*R* ^2^ = 0.2102, *p* = 0.0020). A weaker association between disease resistance and altitude was observed in males (*R* ^2^ = 0.09931, *p* = 0.0396), which fell short of significance in females (*R* ^2^ = 0.07018, *p* = 0.0860). It is conceivable that a population's position relative to the species range, rather than latitude or climatic variables per se, exerts a stronger influence on disease resistance (Woods et al. 2012). We found that disease resistance was greater towards the range center (Ghana) although this association was less pronounced than that of latitude. Notably, populations from low latitudes, such as Monkey Hill (MH), consistently exhibited greater disease resistance regardless of distance from the range center.

Both sexes exhibit increased Ma549 LT_50_ values with annual averages, high and low temperatures at collection sites (Appendix S1: Table A2, Appendix S7C–E). LT_50_ values also correlate with annual mean precipitation (M) and precipitation in the wettest month (W) (Appendix S1: Table A2, Appendix S7F,G, and Materials and Methods). Despite differences in intercepts caused by males being more resistant, regression slopes for males were slightly, but not significantly, steeper, suggesting the sexes were similar in how LT_50_ values trended with changes in temperature and precipitation.

Table 4 presents a summary of the principal component analysis (PCA). The first principal component, accounting for 57.5% of the variance in disease resistance, represents a general gradient of environmental temperature, latitude, precipitation, and relative humidity. The second component, explaining 15.8% of the variance, shows relatively weak correlations with male and female LT_50_ values. The third component, responsible for 10.9% of the variance, delineates the impact of dispersal distance on male disease resistance. According to the eigenvalue greater than 1.0 Kaiser‐Guttman rule, only the first three principal components (Prin 1–3) with eigenvalues greater than 1.0 are retained, as they explain more variance than any individual original variable.

**TABLE 4:** Principal component loadings of environmental variables and LT_50_ for male and female flies.

Principal component analysis (PCA)	Prin1 (57.5%)	Prin2 (15.8%)	Prin3 (10.9%)
T20°C–30°C & RH60‐100%	**0.954346**	*−0.030905*	*0.181506*
T20°C–30°C	**0.921278**	*−0.241807*	*0.224949*
Average temperature (°C)	**0.837885**	**−0.419570**	*0.265887*
Female LT_50_	**0.827146**	*0.214348*	*−0.249415*
Log_10_ (latitude)	**−0.826659**	**0.380707**	*−0.131953*
Male LT_50_	**0.727170**	*0.141419*	−0.401798
Log_10_ (precipitation)	**0.547000**	**0.669968**	*0.132934*
RH60%–100%	**0.622824**	**0.636057**	*−0.031578*
Dispersal distance	**−0.354985**	**0.349947**	**0.751843**

*Note:* Positive or negative loadings indicate a direct or inverse relationship with the principal component. Bold or Italic values denote strong or weak associations between LT_50_ and certain environmental variables, respectively.

### 3.5 Abiotic and Biotic Stresses Link Ecological Pressures to Variations in Disease Resistance

**FIGURE 3:**
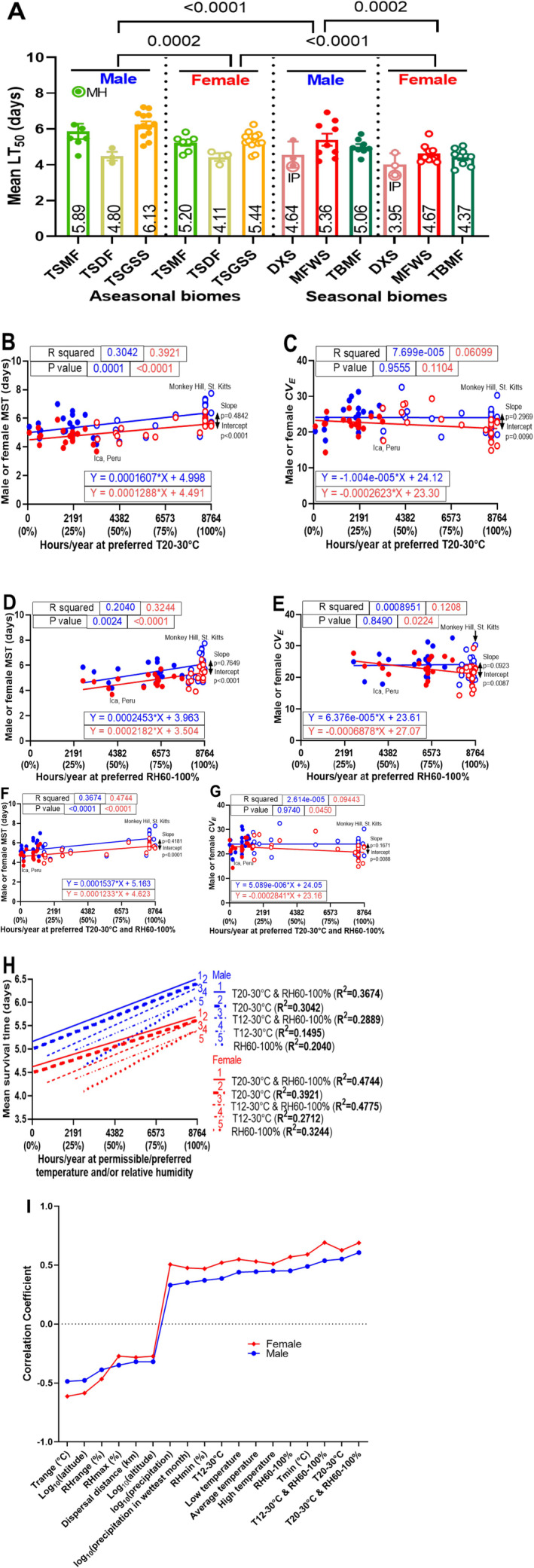
Biome variation in LT_50_ values among 43 global fly lines infected with Ma549 (A). The biome abbreviations and colors for the 43 worldwide fly lines match those in Figure 2A. LT_50_ values varied significantly across biomes and sexes, without a significant biome‐sex interaction, as revealed by a two‐way ANOVA. Significant *p*‐values and LSM ± SEM from Tukey's multiple comparison tests are shown. Males of the highly resistant MH line in the TSMF biome and highly susceptible IP lines in the DXS biome are indicated. Results for the preferred 20°C–30°C temperature range with MST (B) and *CV*_*E*_ (C), preferred humidity with MST (D) and *CV*_*E*_ (E), and the combination of preferred temperature and humidity with MST (F) and *CV*_*E*_ (G), including *R* ^2^ values, *p*‐values, and regression equations with slopes. Male (female) regression lines are blue (red). Aseasonal flies are depicted as circles, and seasonal flies as solid dots; this distinction is consistent in all figures. (H) Comparison of regression slopes (*R* ^2^). (I) Correlation coefficients (*r*) between environmental factors and disease resistance (MST) in male and female *Drosophila* populations included, from left to right, annual temperature range (Trange), latitude, annual relative humidity range (RHrange), annual precipitation, precipitation in the wettest month, annual minimum relative humidity (RHmin), hours/year at 12°C–30°C (T12°C–30°C), low (night) temperature, average temperature, high (day) temperature, hours/year at 60%–100% relative humidity (RH60%–100%), annual minimum temperature (Tmin), hours/year of T12°C–30°C and RH60%–100%, hours/year at 20°C–30°C (T20°C–30°C), and hours/year of T20°C–30°C and RH60%–100%.

Annual temperature and precipitation metrics provide a broad environmental overview but may fail to capture the short‐term variations and extremes that ectotherms must withstand (Manenti et al. 2021; Enriquez et al. 2020). These variations are expected to be most pronounced in seasonal environments. Accordingly, aseasonal biomes (TSMF, TSGSS, TSDF) typically display stable temperatures, high humidity, and plentiful resources, whereas seasonal biomes (TBMF, MFWS, DXS) frequently encounter more extreme environmental stressors. Following infection, aseasonal males and females exhibited longer lifespans than their seasonal counterparts by factors of 1.15 and 1.16, respectively (Figure 3A and Appendix S1: Table A8C,D). The most disease‐resistant lines from Monkey Hill (MH) and Africa were found in the climactically uniform TSMF and TSGSS biomes.

Elucidating biome‐specific factors may reveal the mechanisms by which abiotic and biotic stresses connect ecological pressures to variations in resistance. *D. melanogaster* requires temperatures between 12°C and 30°C and/or relative humidity exceeding 60% for development and fertility (Chakir et al. 2002; Al‐Saffar et al. 1996). The species optimal temperature for activity and development is 25°C (Hamada et al. 2008; Sayeed and Benzer 1996), with temperature fluctuations negatively impacting lifespan (Siddiqui and Barlow 1972). Thus, it was postulated that a narrower temperature range of 20°C–30°C would be more favorable than the broader 12°C–30°C. To examine the impact of time spent outside these thresholds on disease resistance, the average annual exposure (hours per year) within the permissible temperature (12°C–30°C) and preferred humidity ranges (60%–100%) was calculated using NASA Power Data. We analyzed 52,584 hourly temperature and relative humidity data points at the 28 collection sites between 2005 and 2010, corresponding to the collection dates of most of the 43 fly lines (Appendix S1: Table A3). The hours spent per year within the 12°C–30°C range varied from 2263 to 8764 h/year (Appendix S8A–C), while hours at 60%–100% humidity ranged from 2653 to 8764 (Figure 3D,E). Linear regression analyses of the 43 fly populations revealed strong correlations between disease resistance (MST values) and annual hours at 12°C–30°C or 60%–100% humidity for both sexes, showing a trend for increased disease resistance with greater climatic uniformity. Hours within the preferred 20°C–30°C range spanned from 75 to 8764 across collection sites (Figure 3B,C and Appendix S8G) with correlations with disease resistance similar to those for the 12°C–30°C range but exhibiting higher *R* ^2^ values.

The annual duration of concurrent 12°C–30°C temperatures and 60%–100% humidity ranged from 742 to 8764 h across sites (Appendix S8D–F). In contrast, annual hours at 20°C–30°C combined with 60%–100% humidity showed greater variation, spanning from 1 to 8764 h (Figure 3F,G and Appendix S8I). Linear regression analyses of the 43 fly populations revealed that disease resistance in both sexes correlated more strongly with annual hours at 20°C–30°C and 60%–100% humidity than with the 12°C–30°C and 60%–100% humidity combination, or with temperature and humidity considered separately. Male *CV*_*E*_ values showed no correlation with increasing climatic uniformity, whereas female *CV*_*E*_ values exhibited a negative correlation with annual hours at 60%–100% humidity, both with (Figure 3G) and without (Figure 3E) considering annual time at 20°C–30°C. This suggests that consistently favorable humidity conditions do not select for plasticity in females.

**FIGURE 4:**
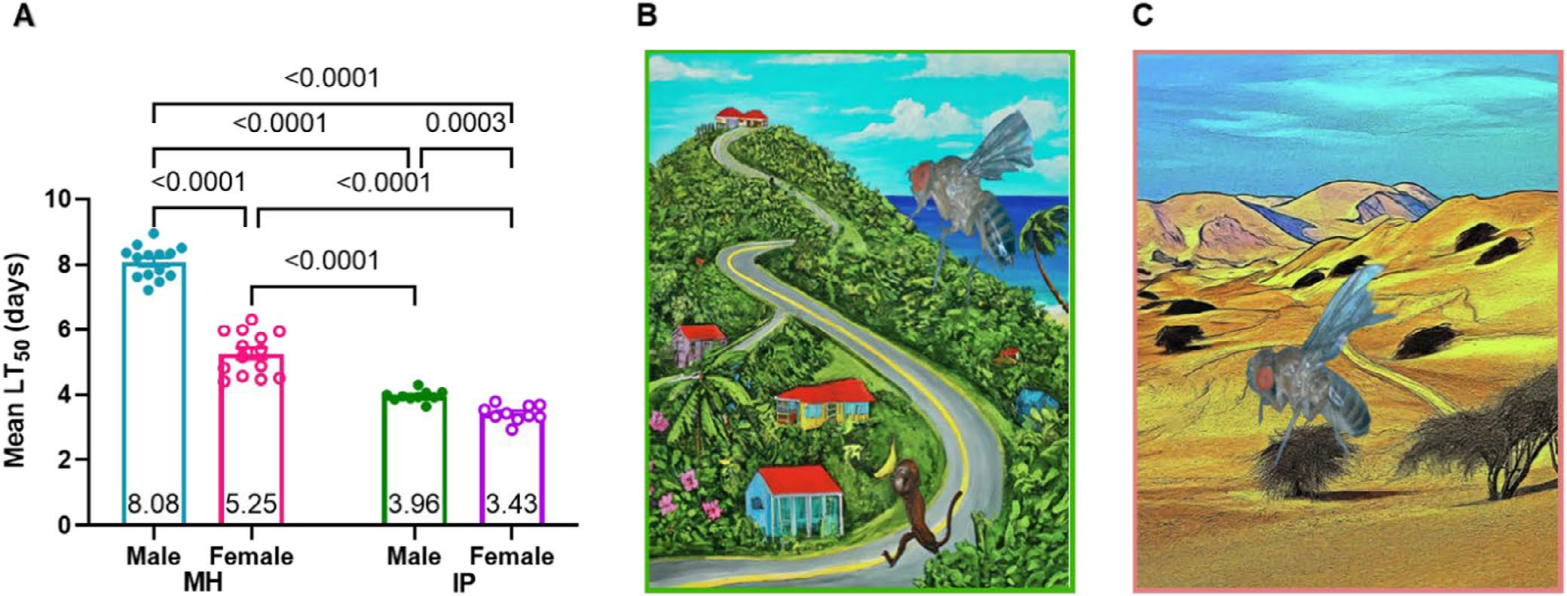
Sexual dimorphism and longevity of infected Monkey Hill, St. Kitts (MH) and Ica, Peru (IP) fruit flies (A): The data present the mean ± SEM LT_50_ values across 14 replicates, with 501 (373) MH females (males) from 3 experiments, and 10 replicates with 281 (339) IP females (males) from 2 experiments. *p* values are derived from Welch's ANOVA tests with Dunnett's multiple comparisons. Cartoon drawings of Monkey Hills, St. Kitts (B) and Ica, Peru (C, contrast their distinct biomes, illustrating the landscapes shaped by each location's unique climatic environment.

We next analyzed populations from the two ends of the resistance and biome spectrum to examine the relationship between abiotic and biotic stresses. The males of the Monkey Hill, St. Kitts (MH) population exhibit the highest disease resistance, whereas males and females of the Ica, Peru (IP) population are the most susceptible (Figures 2B and 4A, Appendix S1: Table A1A,B). MH males and females displayed 2.04‐fold and 1.54‐fold greater resistance to Ma549 infection, respectively, compared to their IP counterparts (*p* < 0.0001, Figure 4A). In relation to the mean LT_50_ values across the 43 strains for males (5.53) and females (4.84), MH males and females demonstrated approximately 1.46‐fold and 1.08‐fold higher resistance, respectively, while IP flies of both sexes exhibited 1.4‐fold lower resistance.

MH in the TSMF biome (Figure 4B) experiences dense vegetation and a consistent maritime climate, whereas IP in the DXS biome (Figure 4C) is subject to arid conditions and sparse desert‐adapted vegetation (Table 1, and Appendix S1: Tables A2, A3). Analysis using Lomb‐Scargle periodograms (Appendix S9A,B, and Appendix S1: Table A3B) confirmed that from 2005 to 2010, MH continuously maintained near‐optimal conditions for fly development (20°C–30°C temperature, 60%–100% humidity). In contrast, IP primarily experienced dry and variable conditions, with favorable temperature and humidity conditions coinciding merely 2.12% of the time (Appendix S9C–J).

**FIGURE 5:**
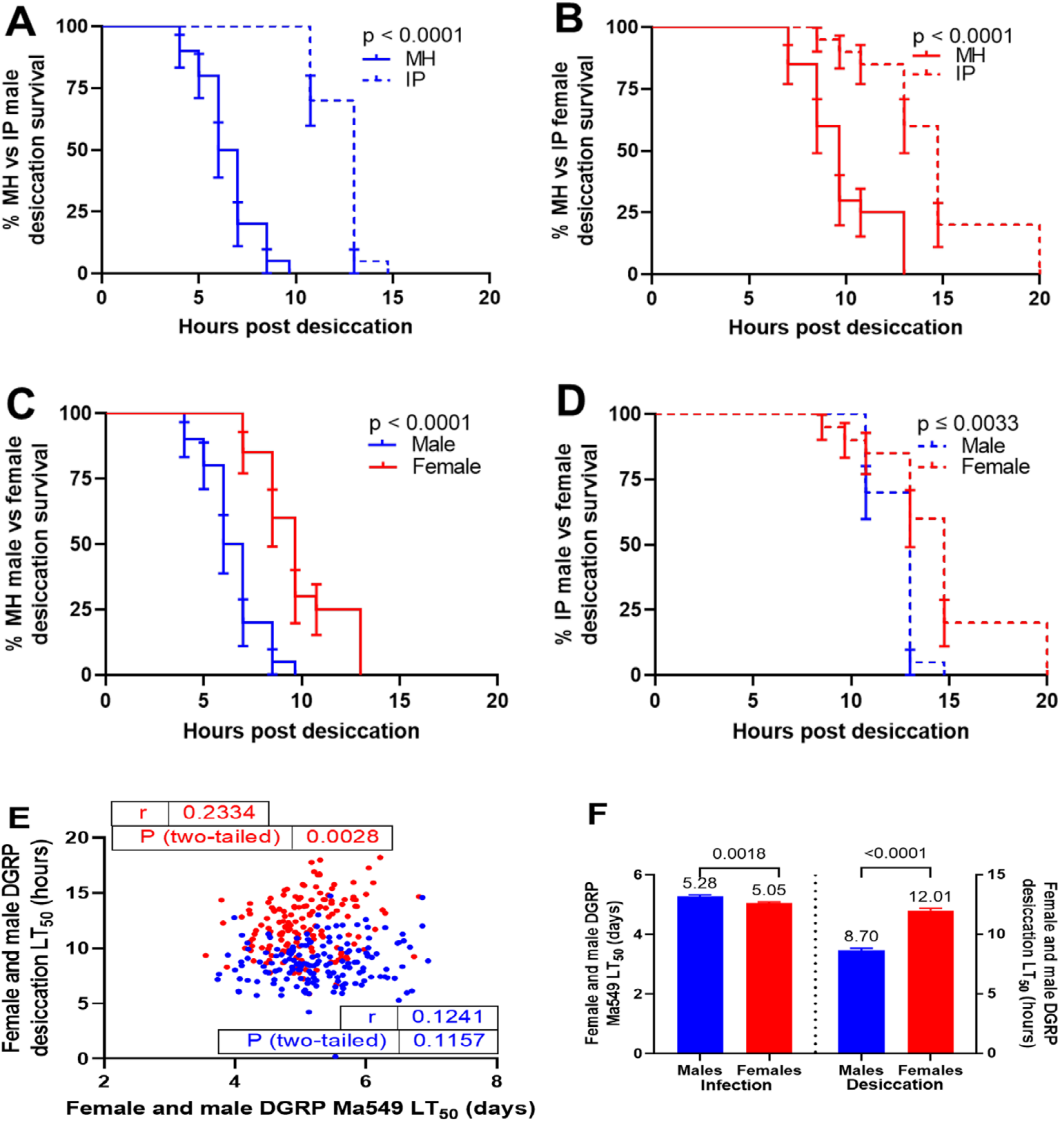
Differences in desiccation resistance. Kaplan–Meier plots illustrate survival differences between MH and IP lines (A, B), and between sexes (C, D). MH males (females) from five replicates (10 flies each) across two trials are shown in turquoise (crimson), and IP males (females) in green (purple). Statistical significance was assessed using log‐rank and Wilcoxon tests. Correlation analysis between desiccation and Ma549 LT_50_ values of 162 DGRP females and males (E), with Pearson correlation coefficients (*r*) and *p* values indicated. Males are represented by blue dots and females by red dots. Sexual dimorphism in resistance to disease (left *y*‐axis) and desiccation stress (right *y*‐axis) for the 162 DGRP fly lines (F) is represented as mean ± SEM LT_50_ values from disease resistance (Wang et al. 2017) and desiccation resistance data (Rajpurohit et al. 2018). *p* values are derived from Welch's *t*‐tests.

We subjected male and female MH and IP flies to a desiccating environment to determine whether there might be trade‐offs with disease resistance. Kaplan–Meier analyses revealed that IP males and females exhibited significantly higher desiccation resistance compared to their MH counterparts, with 2.00‐fold and 1.53‐fold increases, respectively (*p* < 0.0001, Figure 5A,B). Furthermore, females of both lines were more desiccation resistant than males, with IP and MH females outliving their male counterparts by 1.14‐ and 1.49‐fold, respectively (*p* ≤ 0.0033, Figure 5C,D) (Appendix S1: Table A4A). These differences between IP and MH appear adaptive as only IP evolved in a dry environment; however, they totally reverse the direction of resistance to Ma549.

To examine the relationship between disease resistance and desiccation in a larger sample, we reanalyzed disease resistance in the DGRPs (Wang et al. 2017), and the desiccation resistance trait quantified in a later study of the DGRPs (Rajpurohit et al. 2018) (Appendix S1: Table A4B). The analysis revealed a significant correlation between DGRP desiccation resistance and female Ma549 resistance (*r* = 0.2334, *p* = 0.0028), while no such correlation was observed for males (*r* = 0.1241, *p* = 0.1157) (Figure 5E). Moreover, similar to MH and IP, these DGRP lines exhibited a complete reversal in the direction of sexual dimorphism in these two resistance traits: males outlived females by 1.05‐fold in Ma549 resistance, whereas females outlived males by 1.38‐fold in desiccation resistance (Figure 5F).

### 3.6 Correlation Between Sleep and Disease Resistance

Brown et al. (2018) conducted a study on healthy females from 22 global populations, revealing a correlation between higher monthly average temperatures at collection sites and increased sleep duration under laboratory conditions. The current investigation identified a relationship between collection site temperatures and post‐Ma549 infection longevity, while our earlier DGRP analysis linked disease resistance with several sleep parameters (Wang et al. 2017). These findings suggest a potential avenue for exploring the relationship between sleep indices and disease resistance across a global population.

**FIGURE 6:**
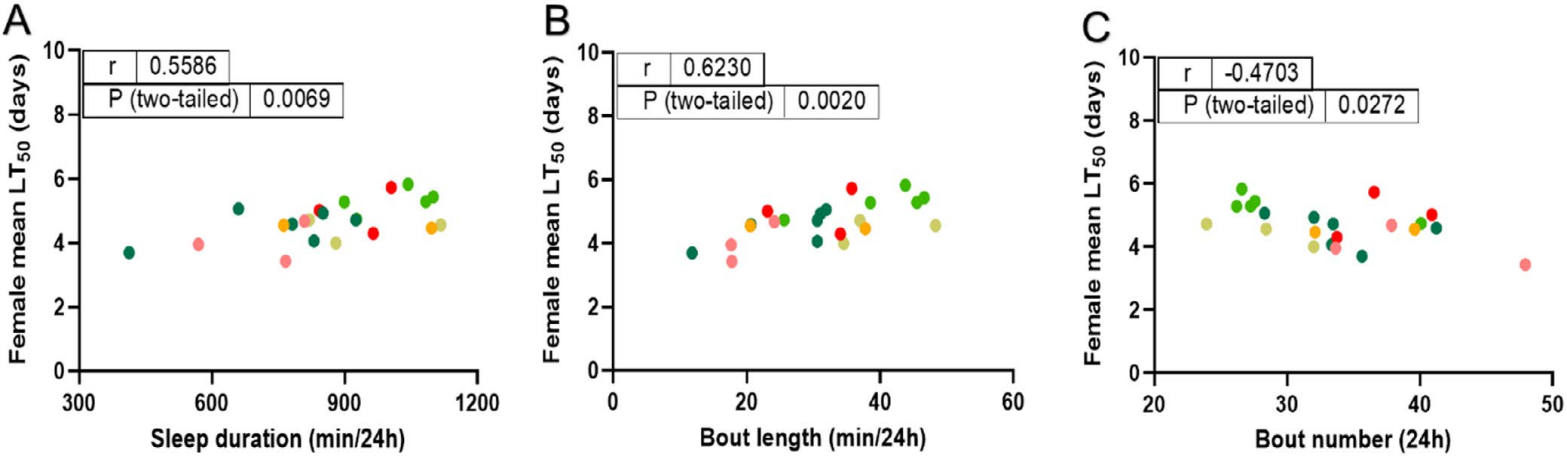
Correlations of LT_50_ values among Ma549‐infected females and publicly available sleep indices for 22 of the lines (Brown et al. 2018) deployed in our study. LT_50_ values are positively correlated with sleep duration (A) and bout length (B), and negatively correlated with bout number (C). Individual Pearson correlation coefficients (*r*) and *p*‐values are indicated in the figures.

Using the sleep behavior data provided for females in 22 global populations (Brown et al. 2018), we identified significant correlations between LT_50_ values and sleep metrics. Positive associations were observed with 24 h sleep duration (*r* = 0.5586, *p* = 0.0069, Figure 6A) and sleep bout length (*r* = 0.6230, *p* = 0.0020, Figure 6B), while a negative correlation was found with sleep bout number (*r* = −0.4703, *p* = 0.0272, Figure 6C). These results underscore a significant link between healthy consolidated sleep patterns and Ma549 infection outcomes.

### 3.7 Characterization of Sickness Sleep Triggered by Ma549 Infection

The significant correlation between healthy female daily sleep patterns and Ma549 infection outcomes may be attributed to two potential factors: the independent influence of latitudinal and climatic gradients on sleep and disease resistance, or an evolutionary interconnection between sleep and resistance mechanisms. To investigate this phenomenon, we examined post‐infection sleep behaviors in MH (resistant) and IP (susceptible) flies utilizing the *Drosophila* Activity Monitor (DAM2) (Shaw et al. 2000) focusing on the initial 2 days post‐infection prior to the onset of overt symptoms. Additionally, we investigated a MFWS population from Crete, Greece (CG). Male and female CG flies displayed LT_50_ values of 4.34 and 4.30 days, respectively (Table 1), with a minimal 0.04‐day difference between sexes (Figures 2C and 9A).

**FIGURE 7:**
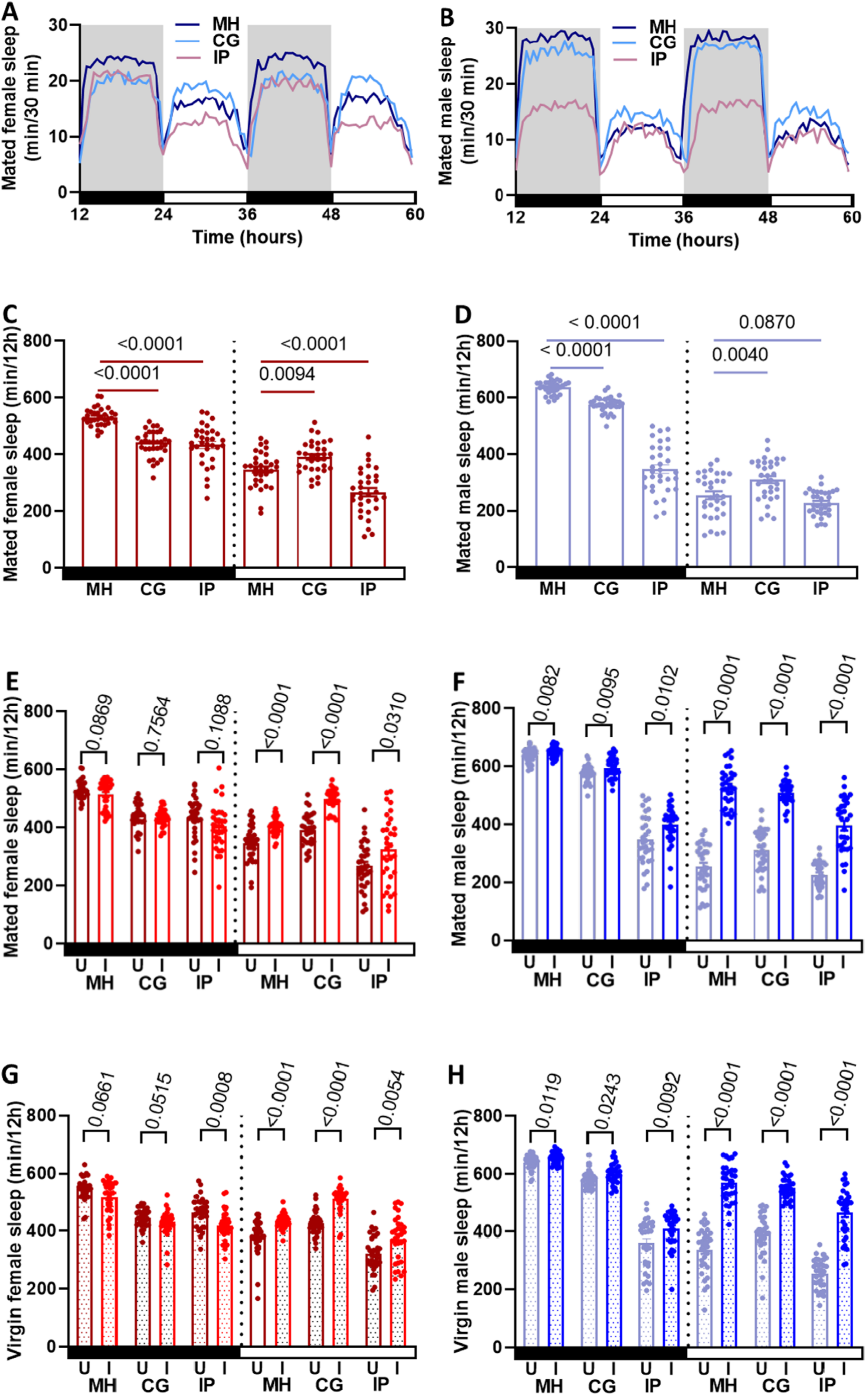
Ma549 infection affects sleep and survival. Sleep profiles depict sleep in min per 30 min from 12 to 60 h spanning two light–dark cycles for 32 flies pooled from three trials for uninfected mated MH, CG, and IP females (A) and males (B), with gray shading indicating nighttime. Sleep comparisons were made between uninfected mated MH versus CG or IP females (C) or males (D), and uninfected (U) versus infected (I) mated females (E), mated males (F), virgin females (G), and virgin males (H) within the MH, CG and IP lines. Bars illustrate nighttime (black) and daytime (white) sleep for uninfected (dark red for females, sky blue for males) and infected (scarlet red for females, navy blue for males) mated and virgin (shaded) flies, with each data point representing the average sleep/12 h of a single fly (*n* = 32) from 12 to 60 h post infection. In panels (C) and (D), means ± SEM and *p*‐values were obtained from one‐way ANOVA. In panels (E)–(H), Mann–Whitney *U* tests, Welch's *t*‐tests, and unpaired Student's *t*‐tests were used for various comparisons, with mean ± SEM and *p*‐values indicated.

Analysis of sleep data (Appendix S1: Table A5) showed that uninfected mated MH females experienced 16% less nighttime sleep but 36% more daytime sleep compared to uninfected males (*p* < 0.0001, Figure 7A–D), which averaged out to females sleeping approximately 12 min less per day. Ma549 infection led to a significant (*p* < 0.0001) increase in daytime sickness sleep (additional sleep following infection, as defined by Davis and Raizen (2017)) without disrupting circadian rhythm. Mated males exhibited the largest increase (107%), followed by virgin males (68%), mated females (17%) and virgin females (14%) (*p* < 0.0001, Figure 7E–G). Nighttime sleep increased by 2% in infected males (*p* ≤ 0.0119) but was unaffected in females (*p* ≥ 0.0661) (Figure 7F,H).

Compared to uninfected MH flies, CG females and males slept less at night and more during the day (Figure 7A–D). CG females slept 24% less at night and 26% more during the day compared to males (*p* < 0.0001, Figure 7A–D). Infection with Ma549 increased daytime sleep in CG flies: mated males by 64%, virgin males by 41%, mated females by 27%, and virgin females by 20% (*p* < 0.0001, Figure 7E–H), with minimal effects on nighttime sleep (Figure 7E–H).

The highly susceptible IP flies had shorter sleep durations than MH or CG flies, with males being more active (slept less) at night (Figure 7A–D). Unlike the MH and CG lines, where males slept longer during the daytime, IP females slept 25% longer at night (*p* < 0.0001, Figure 7A–D) and 18% longer during the day (*p* = 0.0172, Figure 7A–D). Ma549 infection increased daytime sleep in IP flies; mated males by 74% (*p* < 0.0001, Figure 7F), virgin males by 81% (*p* < 0.0001, Figure 7H), mated females by 21% (*p* = 0.0310, Figure 7E), and virgin females by 16% (*p* = 0.0054, Figure 7G), with minimal effects on nighttime sleep (Figure 7E–H).

**FIGURE 8:**
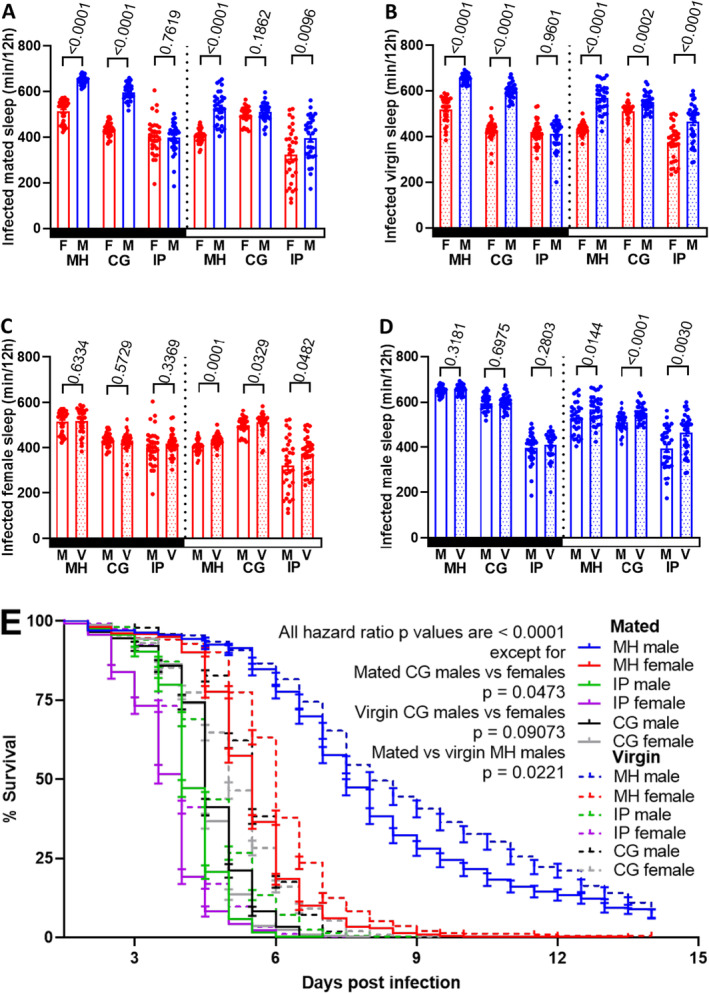
Sexual dimorphism in sleep was compared in mated (A) and virgin (B) MH, CG, and IP flies. Sleep was averaged per 12 h for each fly (*n* = 32) during day and night from 12 to 60 h post infection, pooled from three trials. Mann–Whitney *U* tests, Welch's *t*‐tests, and unpaired Student's *t*‐tests were used for various comparisons, with mean ± SEM and *p*‐values indicated. Variations in nighttime and daytime sleep between mated and virgin infected female (C) and male (D) flies are shown, with each point representing a single fly's average sleep per 12 h (*n* = 32 pooled from three trials) from 12 to 60 h post‐infection. Significance was assessed between mated and virgin females or males within each lines using various tests, with mean ± SEM and *p*‐values indicated. Black (white) rectangles on the *x*‐axis represent nighttime (daytime), and shaded bars denote virgin flies. Line abbreviations: V for virgin and M for mated. Kaplan–Meier plots (E) depict survival differences between female (*n* = 281–585) and male (*n* = 337–434) or between mated (*n* = 281–501) and virgin (*n* = 337–585) flies in each line post‐infection. Statistical comparisons were performed using the Cox Proportional Hazards Mixed Effects model and the emmeans package in R to estimate the hazard ratio, which represents the relative likelihood of death. *p*‐values are provided for both general comparisons and specific group comparisons.

As males are generally more resistant than females, we investigated sexual dimorphism in sleep as a factor in disease resistance (Figure 8A,B, Appendix S1: Table A5). Compared to females, Ma549 infection significantly increased daytime sleep in mated and virgin males of MH (30% and 31%), IP (22% and 24%), and virgin CG (8%) lines (*p* ≤ 0.0096). The effect was minimal in mated CG males (2%, *p* = 0.1862), as was the difference in disease resistance between mated CG males and females. Nocturnal sleep duration in mated and virgin MH and CG males surpassed that of females by 27% and 40%, respectively (*p* < 0.0001), whereas IP males and females showed no significant disparity (*p* ≥ 0.7619). These sleep pattern differences were correlated with disease resistance. We compared the survival of different groups by estimating the hazard ratio (HR), a statistical measure that represents the relative risk of mortality at any given time point over the 14‐day observation period. The hazard ratio for mortality in mated (virgin) females was 5.03 (3.70), 1.15 (1.13), and 1.70 (1.86) fold that of males in the MH, CG, and IP lines, respectively (*p* < 0.0001, Figure 8E, Table 1 and Appendix S1: Table A1C). These findings indicate that, with the exception of virgin CG males, both mated and virgin males exhibited longer sickness sleep and greater disease resistance compared to females post‐Ma549 infection.

Mating status did not affect nighttime sleep of infected females or males across the three strains (*p* ≥ 0.2803, Figure 8C,D and Appendix S1: Table A5C), but sexual activity significantly decreased daytime sickness sleep irrespective of sex and infection status (*p* ≤ 0.0482), with infection reducing daytime sleep by 3%–15% in mated flies compared to virgins. Following infection, the hazard ratio for mortality in virgin females (males) was 0.41–0.61 (0.42–0.84) times that of mated counterparts (*p* < 0.0001, Figure 8E, Table 1 and Appendix S1: Table A1C), demonstrating the substantial impact of sexual activity on sleep regulation and immunity in *Drosophila*, irrespective of genetic background or specific fly lines.

ANOVA was used to validate the effects of genetic line, sex, mating status, and infection on both nighttime and daytime sleep. The results are detailed in Appendix S10 (derived from data in Appendix S1: Table A5B) and visualized in Appendix S11A,B.

### 3.8 The Impact of Host Variation on Fungal Fitness

**FIGURE 9:**
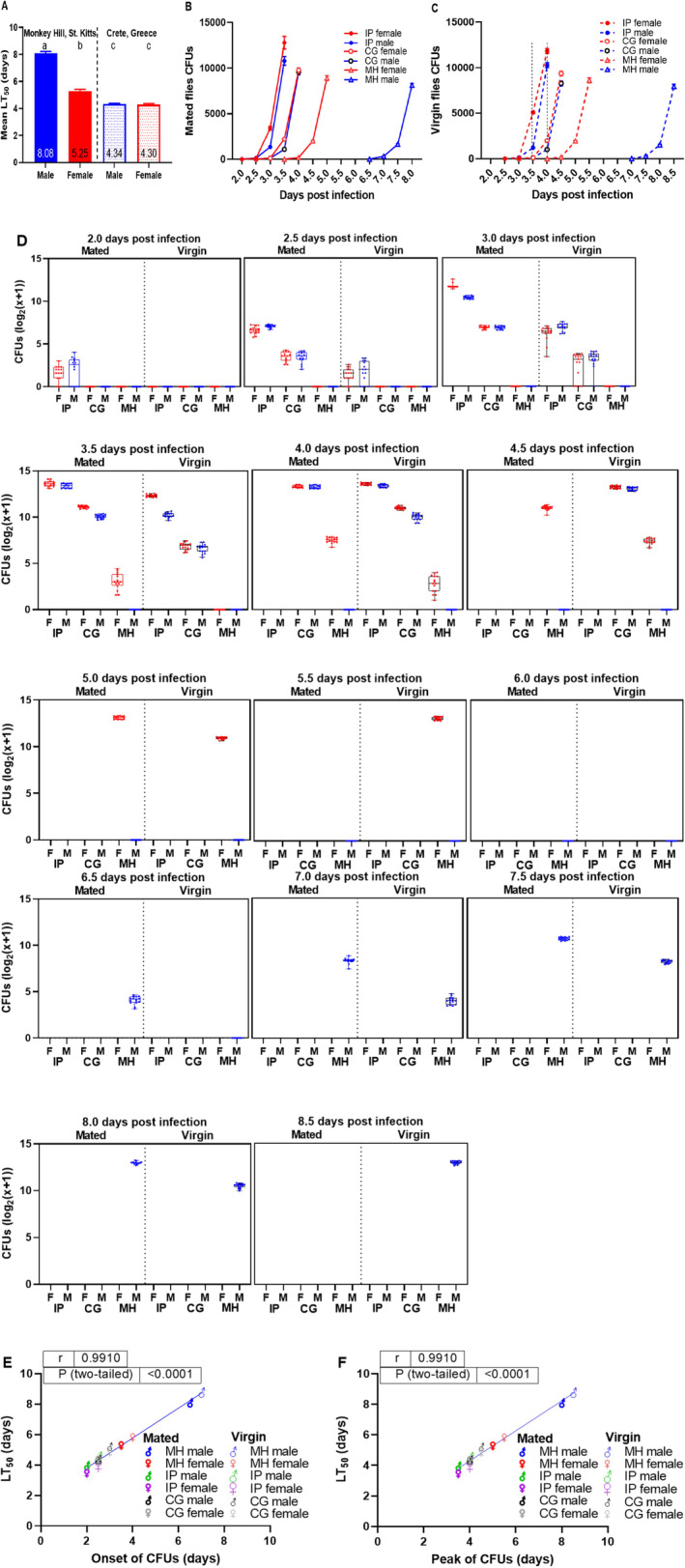
(A) Sexual dimorphism and longevity of infected Monkey Hill, St. Kitts (MH), and Crete, Greece (CG), fruit flies. The data display the mean ± SEM LT_50_ values from 14 replicates, with 501 MH females and 373 MH males across 3 experiments, and 422 CG females and 352 CG males across 2 experiments. Bars not connected by the same letter are significantly different. CFU production time course in mated (B) and virgin (C) CG, IP, and MH flies following Ma549 infection. Each data point represents the average of CFU values from 15 flies. Means ± SEM and *p*‐values were obtained from two‐way ANOVA. Time course of log‐transformed Ma549 fungal loads in the hemolymph of CG, IP, and MH flies following Ma549 infection (D). Box plots depict each time point for each of the 15 individual replicates per sex, mating status, line, and time point across three repeated experiments, showing the mean ± SEM of CFUs. Correlations between LT_50_ values and CFU onset (E) or peak (F) were analyzed for infected mated and virgin CG, IP, and MH females (red) and males (blue). Pearson correlation coefficients (*r*) and *p*‐values indicated.

We analyzed the relationship between longevity and Ma549 colonization (measured as fungal load in colony forming units CFU) of the most resistant (MH), susceptible (IP), and representative average (CG) *D. melanogaster* lines. MH males and females demonstrated significantly extended survival times post‐Ma549 infection, surpassing CG males and females by factors of 2.86 and 2.28, respectively (*p* ≤ 0.0006, Figure 9A). No substantial differences were observed between CG males and females (*p* = 0.9897). Host variation significantly impacted Ma549 fitness, with CFUs appearing 2.0 days post‐infection in IP flies, while appearing at 4.0‐ and 7.0 days post‐infection in virgin MH females and males, respectively. Mated IP females were the least able to restrain fungal growth with 12,791 ± 700.05 CFUs/fly on day 3.5. In contrast, virgin MH males displayed the most effective containment, with only 16 ± 1.21 CFUs/fly on day 7.0 (Figure 9B–D, Appendix S1: Table A6). Fungal loads increased rapidly near fly death in all lines, suggesting a collapse of host resistance mechanisms. Males, regardless of mating status, had lower fungal loads than females (Figure 9B–D), and virgin females delayed fungal proliferation by approximately 0.5 days compared to mated females (Figure 9C,D), aligning with disparities in their LT_50_ values (Figures 4A and 9A, Table 1 and Appendix S1: Table A1C). Correlation analyses revealed a strong association between LT_50_ values (Table 1 and Appendix S1: Table A1C) and both the onset (initial detection of fungal growth) and peak times (maximum fungal growth) of CFUs (*r* = 0.9910, *p* < 0.0001, Figure 9E,F) across all studied groups.

These findings substantiate the influence of fly line, sex, and mating status on fungal invasiveness, with proliferation coinciding with and likely causing death due to the host's inability to tolerate high fungal levels (Figure 9).

### 3.9 Potential Adaptive Differences Between Populations From Africa, France, and Raleigh, North Carolina

Based on the observed longevity differences across geographic regions and biomes, we explored the underlying genetic basis by analyzing single nucleotide polymorphisms (SNPs) linked to disease resistance. Using SNPs from the North American DGRP (Wang et al. 2017), we investigated allele frequency differences that could indicate local adaptation to pathogens in the DGRP, African, and French isolates (Appendix S1: Table A7). We previously found that most polymorphisms associated with Ma549 resistant DGRP lines were in the lower range of the allele frequency spectrum, with frequencies below 0.2 for 41% of the genes. These low‐frequency alleles had larger effects on LT_50_ values than common alleles, resulting in the most Ma549 resistant DGRP lines having a preponderance of these alleles (Wang et al. 2017). Given that African lines typically exhibit higher resistance than DGRP lines and are localized in an aseasonal biome (Appendix S5), we hypothesized that these alleles may be more prevalent in Africa.

We defined subsets of rare SNPs (Appendix S1: Table A7) previously linked with quantitative traits (LT_50_ values or *CV*_*E*_, micro‐environmental plasticity as described (Morgante et al. 2015)) in Ma549 resistant DGRP lines using their corresponding positions in African and European populations. We used the PopFly database (Hervas et al. 2017) to download genome sequences from African lines corresponding to the 10‐bp region flanking each SNP. Besides the 10 lines from Ghana, we included the Siavonga, Zambia (ZI) population, which consists of 207 individuals (large sample size) and is the most diverse among all sampled populations, but with minimal admixture from non‐African populations (Lack et al. 2016; Hervas et al. 2017).

**FIGURE 10:**
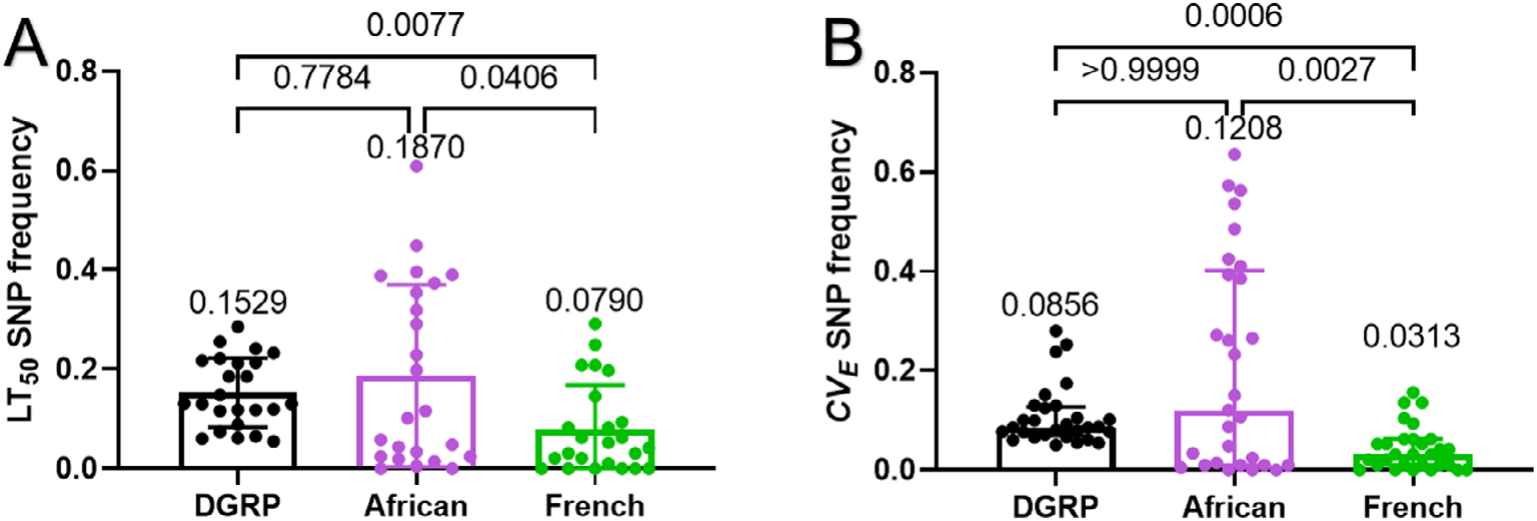
Frequency analysis of single‐nucleotide polymorphism (SNP) for LT_50_ (A) and coefficient of variation (*CV*_*E*_) (B) among DGRP, African, and French populations. Welch's ANOVA tests (*W*_(2,42.24)_ = 6.289, *p* = 0.0041) reveled significant differences in LT_50_ SNP frequency across the DGRP, African, and French groups. Dunnett's multiple comparisons test identified significant *p*‐values and mean ± SEM (A). The Kruskal–Wallis test showed significant differences (*H* = 16.58, *p* = 0.0003) among the groups, with significant *p* values and median ± IQR from Dunn's multiple comparisons test noted (B).

The African populations exhibited higher frequencies of 11 out of 24 alleles affecting LT_50_ values in the DGRP lines. Variants typically showed modest two‐ to threefold differences between African and American populations, but some SNPs displayed approximately 10‐fold differences, mostly favoring African populations, resulting in a 22.3% higher average frequency of resistance alleles in African than in American lines (Figure 10A). Sixteen out of 29 alleles influencing *CV*_*E*_ values were more prevalent in African lines, with frequency differences leading to twice as many *CV*_*E*_ variants for high plasticity in African genomes than in the DGRP lines (Figure 10B). Low frequency (< 0.08) DGRP variants in *Rab26* (exocrine secretion), *Xpd* (DNA repair, UV damage) and at an intergenic site were the major alleles (> 0.5) in African populations, whereas DGRP variants in two poorly understood genes, *CG32066* (DGRP frequency 0.1311) and *CG13229* (DGRP frequency 0.2327), were absent in Zambia and Ghana populations. An extended search in the PopFly database across additional African populations (136 Cameroon, 680 Ethiopia, 136 Gabon, 176 Kenya, 88 Malawi, 88 Nigeria, 136 Rwanda, 504 South Africa, 176 Uganda and 88 Zimbabwe) revealed the DGRP SNP variant of *CG32066* in single flies from South Africa (SD39N) and Uganda (UG19), but the DGRP *CG13229* variant was not found in Africa.

The DGRP is an admixture of African and European populations (Pool 2015). We extended our search to 172 French genomes in PopFly, finding that seven of the DGRP SNPs were absent. Most SNPs, except for those in *gem3, ash2, CG33136, Ddr, CCKLR‐17D3*, and *lar*, segregated at < 0.1. All sites had missing data (> 17%, < 96%) across the French genomes. Conversely, 34 out of 59 DGRP SNPs in the Zambian population had no missing data, and nine SNPs had less than 10% missing data. We also examined whether DNA near the SNPs showed adaptive functional differences between the DGRP and other populations using the *F*_ST_ (fixation index) estimator embedded in PopFly. We estimated *F*_ST_ values for the one kb sequence surrounding SNPs with a minor allele frequency higher than 5% in DGRP and Zambian populations. The Ethiopian population was included as another ancestral African population. Mean pairwise comparisons yielded *F*_ST_ values of 0.154 ± 0.0153 and 0.179 ± 0.0292, respectively, between DGRP and Zambian or Ethiopian populations, respectively. There was approximately threefold less differentiation between the American and French populations (*F*_ST_ = 0.0461 ± 0.01304, *z* = 4.67399, *p* < 0.00001, Mann–Whitney *U* test), showing that despite greater conservation of resistance‐associated SNPs with ancestral African populations, the DGRP shows less population differentiation with French than with ancestral African populations in the surrounding DNA regions.

This is surprising since North American populations contain African and European DNA, but it aligns with comparative studies on microsatellites that showed mean pairwise comparisons between African and non‐African populations produce high *F*_ST_ values ranging from 0.11 to 0.20, while European and American populations have average pairwise *F*_ST_ values of 0.071 (Caracristi and Schlötterer 2003).

## 4 Discussion

This study extends previous research on disease resistance variation in the DGRP from North Carolina, United States, to encompass global populations reflecting the evolution of *D. melanogaster* as a human commensal. The investigation utilized 43 global stocks established at different times, acclimated in laboratory conditions and assayed under standardized laboratory parameters. The findings revealed persistent latitudinal clines for disease resistance based on the original collection sites of these stocks. A limitation of this research is the potential for laboratory adaptation and inbreeding effects on the lines. However, this may not significantly impact the results, as previous studies have demonstrated that laboratory‐maintained inbred *Drosophila* populations exhibit similar trait correlations and plasticity in morphological traits and stresses as freshly caught populations (Maclean et al. 2018), which is consistent with expectations for clinal traits shaped by natural selection (Souto‐Maior et al. 2020). Furthermore, we found no evidence of a relationship between stock age and disease resistance in our data. We also took the precaution of testing multiple stocks to provide sufficient independent data points. Finally, as noted by other researchers using laboratory stocks to study clinal signals (Fabian et al. 2015), if laboratory adaptation is present, it would likely act against or potentially eliminate the clinal signals detected in this study.

Our data suggest a correlation between disease resistance and collection sites in close proximity to the equator with higher average annual temperatures, as described previously for sleep (Brown et al. 2018). We added 21 lines to the 22 used by Brown et al. (2018), incorporating African populations to provide representation from near the ancestral range of the species (Accra, Ghana). These Ghanian lines, which cluster with populations from Zimbabwe and Zambia, contributed to the discovery that sub‐Saharan populations are more diverse than other populations (Pool 2015; Lack et al. 2016; Verspoor and Haddrill 2011). Accra's equatorial location within the TSGSS biome results in minimal fluctuations in seasonal temperature and humidity (96% of the year falling within the preferred 20°C–30°C temperature and 60%–100% humidity range), closely resembling the stable, aseasonal Monkey Hill environment. An alternative explanation to latitudinal clines posits that distance from the range center, regardless of direction may be associated with altered species interactions and reduced genetic diversity, which may translate to reduced disease resistance. This “range center” hypothesis suggests that distance from Africa should negatively correlate with disease resistance to a comparable degree as latitudinal clines. However, it does not despite latitude being a component of distance. The tendency of lower latitude flies to exhibit higher resistance persisted over large distances suggesting that latitude or climatic variables are more reliable predictors of disease resistance than a population's relative position within the species range.

Given the African origin of *D. melanogaster* , the non‐African population diversity is expected to be a subset of African diversity (Sprengelmeyer et al. 2019). However, 12 (13) of the 15 African male (female) lines ranked among the 50% most resistant. Exceptions were observed in flies from Madagascar and Zimbabwe and male flies from Malawi, regions characterized by significant seasonal fluctuations in temperature and especially humidity. This indicates that the genomic diversity of African populations did not translate into phenotypic diversity in resistance to Ma549, as they were generally resistant, possibly due to adaptation to aseasonal habitats. Similarly, four African *D. melanogaster* lines were more resistant to the fungal pathogen *Beauveria bassiana* than two non‐African populations (Tinsley et al. 2006). The authors postulated that if similar variations in pathogen susceptibility exist in pest insects, it could potentially lead to the evolution of resistance against fungi developed as biopesticides (Tinsley et al. 2006). Notably, the frequency of SNPs associated with resistance to Ma549 in the DGRP was intermediate between African and European genomes, despite the conservation of surrounding DNA regions with Europeans. This discrepancy might be due to the preferential selection of African SNPs in the DGRP or even the occurrence of *de novo* mutations in America, as most were rare or absent in Europe.

African populations, as well as other aseasonal (tropical) populations from uniform climates, such as MH, generally demonstrate superior disease resistance compared to seasonal (temperate) populations that endure intermittent thermal and humidity stress. Relatively little is known about the evolution of clines governed by interspecific interactions. However, the differences we found between *Drosophila* populations are aligned with studies linking climate variables to malaria risk. Specifically, uniform temperatures and high humidity support *Anopheles gambiae* vector populations and influence malaria transmission patterns (Paaijmans et al. 2009), underscoring the importance of aseasonal regions in disease dynamics. Moreover, *A. gambiae* populations exhibit extensive clinal variation along an aridity gradient (Cheng et al. 2012). *D. melanogaster* thrives within an optimal temperature range crucial for activity and development (Hamada et al. 2008; Sayeed and Benzer 1996), while temperature fluctuations can negatively impact its lifespan (Siddiqui and Barlow 1972). Additionally, relative humidity above 60% significantly enhances fly longevity, particularly in females (Tochen et al. 2016). Several studies have reported increases in *Drosophila* populations correlating with rainfall (Achumi et al. 2013; Bombin and Reed 2016), and fungal insect diseases, including Ma549, are strongly favored by humid conditions (Wang et al. 2023), suggesting a link between precipitation and increased disease exposure. Aseasonal regions characterized by stable climates and abundant food resources harbor most terrestrial biodiversity and species interactions (Brown 2014; Salazar‐Mendoza et al. 2021). The greater species richness and microbial prevalence in these areas may drive the evolution of enhanced host defenses (Schemske et al. 2009). This ecological scenario, featuring concurrent increases in fungal infestations and conditions favorable to fungal infections, may exert more intense selective pressure for disease resistance at lower latitudes. Analogously, genetically based plant defenses against herbivory are higher at lower latitudes corresponding to greater levels of herbivory in the tropics (Pennings et al. 2009).

Resistance to abiotic stress is likely to be a larger component of fitness at high latitudes. Stress responses are metabolically costly, and surviving abiotic stresses may require nutrient allocation that potentially compromises immune function (Landis et al. 2012). Consequently, the adaptive processes to abiotic stresses crucial for the persistence of temperate populations may result in a trade‐off against alleles that confer greater resistance to disease. This concept aligns with Hoffmann et al.'s proposed evolutionary trade‐off, wherein individual flies cannot possess both cold resistance and starvation resistance, with the latter being selected for in warm climates (Hoffmann & Hoffmann, 1999; Hoffmann et al. 2005). Our previous DGRP analysis identified an association between starvation resistance and disease resistance (Wang et al. 2017), indicating that it could be one of the factors contributing to adaptive differences in disease resistance. Starvation resistance, linked to metabolic storage and homeostatic maintenance, may facilitate coping with both abiotic and biotic stressors. However, high resource availability and pathogen prevalence in tropical climates could also select for disease resistance. A separate DGRP investigation uncovered significant correlations between abdomen size at 28°C and chill coma recovery as well as between abdomen size at 17°C and resistance to Ma549 (Lafuente et al. 2018). These findings suggest that disease resistance is linked to developmental plasticity that assists flies in coping with environmental heterogeneity. Most of the variation in the DGRP that predicted disease resistance affected the general robustness of the flies, particularly genes involved in development, nutrition, and sleep patterns (Wang et al. 2017).

Females exhibit a greater dependence on relative humidity values above 60% compared to males (Tochen et al. 2016). Our investigation leveraged previously acquired desiccation and disease resistance data from DGRP lines (Wang et al. 2017; Rajpurohit et al. 2018) to demonstrate a significant link between these traits in females. This correlation suggests cross‐resistance to multiple stresses rather than trade‐offs, implying shared mechanisms underlying some of the observed variation in these traits. A reviewer suggested that we discuss the characteristics of the host cuticle that may influence disease trade‐offs. Intraspecific variation in desiccation resistance in *D. melanogaster* is partially attributed to differences in cuticular hydrocarbon (CHC) composition, with females generally possessing slightly elevated CHC levels compared to males as a desiccation defense mechanism (Wang et al. 2022). Furthermore, these CHCs may impact the surface microbiome, which offers some protection against entomopathogens (Hong et al. 2022). The MH flies present an ideal model for investigating the cuticle's role, given the pronounced differences between males and females; nonetheless, our current findings suggest a post‐penetration disparity between male and female flies in suppressing colonization. The observed correlation between the resistance of DGRP lines to the bacterium *Pseudomonas aeruginosa*, the narrow host range *Entomophthora muscae* (Entomophthoromycota) and Ma549 indicates the presence of general (non‐specific multipurpose) defense mechanisms, suggesting that resistance to one pathogen is not traded off with increased susceptibility to another (Wang et al. 2017, 2020).

The hypothesis that disease resistance is associated with adaptation to local environments is supported by observations of desiccation resistance, where laboratory conditions that simulate the low humidity of IP favor IP flies over MH flies. However, if certain insects have evolved an increased generalized resistance to multiple stressors, then resistance to Ma549 may be a coincidental side effect of cross‐resistance to other stressors rather than a result of evolutionary interactions with pathogens. The fly's immune system is known to have functions beyond defense against pathogens, with cold stress in particular known to activate immune genes and increase resistance to *B. bassiana* (Wiil et al. 2023). Likewise, reactive oxygen species trigger certain innate immune mechanisms (Ramond et al. 2021), and also sleep, which protects against oxidative stress (Hill et al. 2018). Consequently, it is not surprising that the resistance of DGRP lines to oxidative stress correlates with resistance to Ma549 (Wang et al. 2017).

It remains unknown whether this multi‐stress resistance is still advantageous when insects simultaneously encounter abiotic and biotic stresses. This scenario is most likely to occur in seasonal regions due to arid or thermal stresses. Although desiccation and disease resistance are associated in females, disease resistance peaks in stable regions where desiccation does not pose a major threat. Flies from IP and males from MH represent the range of variation in disease resistance. Monkey Hill's stable, warm, near‐optimal temperature and humidity (marine ecoregion) presumably imposes less abiotic stress than the seasonally variable, drier desert climate of IP (DXS biome), while pathogen pressure is likely to be low in an arid desert. Relaxation of selective pressure could have led to degeneration of immune defenses in IP flies, similar to *D. sechellia* that lives in an “enemy‐free space” (O'Malley et al. 2023). Conversely, IP flies demonstrate high desiccation resistance, with the highly disease‐resistant MH males being the most susceptible to desiccation . This reversal in the direction of sexual dimorphism aligns with observations from the DGRP lines (Figure 5E,F) and a recent study on sexual dimorphism in 15 *Drosophila* lines, which revealed that males generally exhibit longer lifespans, while females demonstrate greater resistance to starvation and desiccation, with substantial inter‐line variation in these traits (Lin et al. 2023). These findings suggest that the relationship between desiccation and disease resistance is prioritized differently in males and females.

The association between African populations and DGRP was found to be particularly strong for SNPs linked to the phenotypic micro‐environmental plasticity of DGRP lines. DGRP lines exhibit significant variability in their plasticity to disease (Wang et al. 2017) and thermal stress (Lafuente et al. 2018), demonstrating that even within a local population, genotypes can produce flies with different plasticity. Plasticity is generally costly and only selected for in heterogenous environments (Van Den Heuvel et al. 2013), which suggests that males, which typically exhibit greater variation in disease resistance, may be more adaptable or experience a greater diversity of pathogens. Using *CV*_*E*_, we found that selection favors the plasticity of disease resistance in males equally in seasonal and aseasonal populations, whereas in females, there was a trend for reduced plasticity in stable climates. This suggests that aseasonal females may be less resilient to changes in disease exposure. Female fitness is more critical for population persistence and growth than male fitness (Harts et al. 2014), so these differences may affect species survival when confronted with global climate change and infectious diseases (St. Leger 2021).

A central problem in infection biology is determining why two individuals exposed to the same pathogen have different outcomes. We found that the resistant MH, the highly susceptible IP, and a line (CG), which shows almost no sexual dimorphism in disease resistance, had marked differences in their ability to control Ma549 growth and colonization rate, and thus pathogen invasiveness. As variation in survival is linked to fly robustness and resistance to stressors, as well as physiology and behaviors such as sleep, it was anticipated that flies would differ in their ability to tolerate extensive colonization. However, all lines showed low fungal loads until the day before death, suggesting that they die at a specific fungal load. Thus, variation in survival stems from variations in the ability to repress fungal growth, which is most likely determined by the immune system rather than the ability to tolerate extensive colonization. In contrast to anti‐viral defense genes that appear to be rapidly evolving, there is less evidence of local adaptation of immune genes to bacteria and fungi (Early et al. 2017). However, latitudinal clines in polymorphism frequency in the Diptericin gene determine resistance to infection by a bacterial pathogen (Hanson et al. 2019) and there are functional polymorphisms in the Bomanin genes (Smith et al. 2023), which is relevant to our study as Bomanins are the only *Drosophila* anti‐microbial peptides (AMPs) thus far shown to have efficacy against Ma549 (Wang et al. 2023). Classical virulence theory suggests that the extra resistance demonstrated by MH could drive selection for higher pathogen virulence, while tolerance, if it had occurred, could have led to eventual pathogen‐host co‐existence (Howick and Lazzaro 2014).

Using sleep data for healthy females from 22 lines (Brown et al. 2018), we demonstrated that variation in disease resistance was positively correlated with consolidated sleep (sleep duration and bout length) and negatively correlated with fragmented sleep (bout number). This suggests that flies that sleep more while healthy are better at resisting infection. In addition to the diurnal rhythm sleep observed in healthy animals, *Drosophila* increases sleep in response to infection, akin to rabbits reacting to fungal infection (Toth et al. 1993). This “sickness sleep” occurs prior to symptoms such as reduced feeding and upregulation of the immune gene *Drosomycin* during Ma549 infection (Lu et al. 2015; Wang et al. 2023). Our sleep analysis showed that Ma549 infection led to increased daytime sickness sleep in MH, CG, and IP flies, particularly in males, with MH males, who succumbed the slowest to Ma549, showing the most pronounced effect. While the benefits of sleep for diseased organisms remain unclear, a GWAS using the DGRP identified overlapping alleles affecting resistance to Ma549 and sleep, with some alleles exhibiting sex‐dependent effects in sleep and/or disease resistance (Wang et al. 2017). The characterization of NEMURI, an AMP that regulates sleep and bolsters resistance to infection (Toda et al. 2019), provides mechanistic evidence for the observed correlations between sleep patterns and infection resistance, further contextualizing our results. The observed differences between male and female MH, CG, and IP flies may represent a phenotypic manifestation of this phenomenon. Males exhibited a lower fungal burden compared to females, consistent with sex‐specific immune investment strategies. The genetic architecture of many traits, including immunity, differs between male and female *Drosophila* (Belmonte et al. 2019). Moreover, the immune sexual dimorphism common in *Drosophila* lines is dependent on the specific interactions of each pathogen with immune pathways, with certain components differentially affecting male survival and others affecting female survival (Wang et al. 2023). Sexual activity was found to reduce sleep and the ability to delay fungal growth in both sexes, consistent with trade‐offs between reproduction and immunity (Garbe et al. 2016; Rai et al. 2023; Gordon et al. 2022), despite males not experiencing the energy drain associated with egg production (Mishra et al. 2023).

The diverse mechanisms involved in disease resistance have typically been studied independently, limiting our understanding of the interplay between biotic and abiotic stress responses and their relation to disease resistance and ecology. This issue is particularly relevant for clines in sleep, stress resistance, and defense, as these traits are closely linked to fitness. There is growing recognition that stress and defense traits are interconnected with each other and with sleep; however, the general issue of their joint clinal evolution remains unresolved. Given the anticipated climate change and the significance of rapid evolution in invasive species, this is likely to change, and the *Drosophila*‐Ma549 model appears to be well‐suited for these studies. Sleep and stress responses may provide an evolutionary mechanism for optimizing host defenses, but it remains unclear whether selection primarily shapes host defenses through changes in non‐specific stress responses and variations in physiological and behavioral traits such as sleep or, conversely, whether the selection is on stress responses or sleep with disease resistance as a secondary factor. Despite its status as a model system, little is known about *D. melanogaster* behavior in the field (Dukas 2020). Increased daytime activity by healthy MH males may increase sexual success due to the wide dispersion of abundant food resources and females in tropical zones, while tropical females may be less inclined to leave an attractive feeding and oviposition site. Male flies from temperate regions may be less active due to trade‐offs with abiotic stresses, finding shelter near food sources to mitigate environmental conditions (Simon et al. 2011). IP females exhibit greater desiccation resistance than males, but the high nocturnal activity (low sleep) of IP males might feasibly be a behavioral adaptation to their arid environmental conditions and unrelated to disease resistance.

The findings of our study underscore the pivotal influence of environmental factors on fly lifespan post‐infection, highlighting the intricate interplay between ecological context and disease resistance. Within the framework of climate change, increasing environmental variability may have far‐reaching impacts on global disease resistance patterns. Notably, tropical hosts with greater broad‐spectrum disease resistance may be less equipped to adapt to shifting environmental conditions, while tropical pathogens expanding their ranges into new territories will encounter more susceptible populations. This phenomenon carries significant implications for pest management and conservation efforts. Increasing temperatures, altered precipitation patterns, and increased frequency of extreme events under climate change threaten the persistence of many insects and their pathogens (St. Leger 2021). Consequently, understanding the adaptive responses to these environmental stressors is crucial for accurately forecasting climate change impacts (Kellermann et al. 2018).

## Author Contributions

**Mintong Nan:** conceptualization (equal), data curation (lead), formal analysis (lead), investigation (lead), methodology (equal), software (lead), supervision (supporting), validation (equal), visualization (lead), writing – original draft (equal), writing – review and editing (equal). **Jonathan B. Wang:** data curation (supporting), formal analysis (supporting), investigation (supporting), methodology (equal), writing – original draft (supporting), writing – review and editing (supporting). **Michail Siokis:** formal analysis (supporting), investigation (supporting), methodology (supporting), validation (supporting), writing – original draft (supporting), writing – review and editing (supporting). **Raymond J. St. Leger:** conceptualization (equal), formal analysis (supporting), funding acquisition (lead), methodology (equal), project administration (lead), resources (lead), supervision (lead), validation (equal), visualization (supporting), writing – original draft (equal), writing – review and editing (equal).

## Conflicts of Interest

The authors declare no conflicts of interest.

## Supporting information


Appendix S1.

## Data Availability

All relevant data are within the paper and its Supporting Information files.
