# Supplementary material for: Latitudinal Clines in Climate and Sleep Patterns Shape Disease Outcomes in Drosophila melanogaster Infected by Metarhizium anisopliae
Source: Ecol Evol. 2025 Feb 28;15(3):e71047. doi: 10.1002/ece3.71047 (PMC11868735; doi:10.1002/ece3.71047)
Supplement: Supplementary file 1 — Appendix S1. [file ECE3-15-e71047-s001.zip › ece371047-sup-0001-Supinfo1.docx]

# Supplementary files

**Appendix 1: Table A1** Survival data following Ma549 infection for 43 global mated females and males, as well as virgin CG, IP, and MH females and males.

**Appendix 1: Table A2** Monthly average, high, and low temperatures (°C), precipitation and precipitation in wettest month at the collection year of each of 43 lines, along with the ncPick script.

**Appendix 1: Table A3** Hourly temperature and relative humidity data from the years 2005-2010 for 28 locales or 43 fly strains.

**Appendix 1: Table A4** Desiccation data for IP and MH strains, along with disease and desiccation resistance values in 162 DGRP lines in Figure 5.

**Appendix 1: Table A5** Sleep data in Figures 7-8.

**Appendix 1: Table A6** CFU data in Figure 9B-D.

**Appendix 1: Table A7** LT_50_ and CV_E_ SNP frequency in Figure 10.

**Appendix 1: Table A8** ANOVA summaries on main effects and interactions.

# APPENDIX 2

**Comparison of mean and variation in disease resistance between males and females**

| **Line** | **t-test  for equality of means** | | | **Longevity *CV_E_*** | | | **Levene's test for equality of variances** | |
| --- | --- | --- | --- | --- | --- | --- | --- | --- |
|  | **t** | **p value** | **Δmean** | **Male** | **Female** | **Δ*CV_E_*** | **F ratio** | **p value** |
| Accra, Ghana.163 | 9.01 | <0.0001 | 0.98 | 24.99 | 19.71 | 5.28 | 27.51 | <0.0001 |
| Accra, Ghana.165 | 9.41 | <0.0001 | 0.94 | 24.54 | 21.69 | 2.85 | 13.62 | 0.0002 |
| Accra, Ghana.167 | 9.41 | <0.0001 | 0.84 | 21.06 | 17.43 | 3.63 | 26.86 | <0.0001 |
| Accra, Ghana.169 | 6.72 | <0.0001 | 0.86 | 25.42 | 22.99 | 2.43 | 15.63 | 0.0001 |
| Accra, Ghana.171 | 11.44 | <0.0001 | 1.33 | 23.11 | 22.22 | 0.90 | 18.22 | <0.0001 |
| Accra, Ghana.173 | 13.30 | <0.0001 | 1.46 | 25.54 | 17.20 | 8.34 | 72.48 | <0.0001 |
| Accra, Ghana.175 | 6.17 | <0.0001 | 0.68 | 23.75 | 19.38 | 4.37 | 17.27 | <0.0001 |
| Accra, Ghana.177 | 6.17 | <0.0001 | 0.58 | 24.94 | 14.91 | 10.03 | 45.92 | <0.0001 |
| Accra, Ghana.179 | 12.38 | <0.0001 | 1.44 | 26.41 | 19.69 | 6.72 | 41.95 | <0.0001 |
| Accra, Ghana.181 | 1.96 | 0.0500 | 0.19 | 18.87 | 23.50 | -4.63 | 0.93 | 0.3352 |
| American Samoa | 2.36 | 0.0184 | 0.21 | 19.30 | 23.05 | -3.75 | 0.72 | 0.3953 |
| Athens, Greece | 9.02 | <0.0001 | 1.00 | 25.33 | 23.25 | 2.08 | 11.56 | 0.0007 |
| Bahia, Brazil | 1.08 | 0.2816 | 0.09 | 24.14 | 27.99 | -3.84 | 0.02 | 0.8801 |
| Bermuda | -1.66 | 0.0980 | -0.14 | 23.78 | 29.36 | -5.58 | 22.95 | <0.0001 |
| Blacksburg, Virginia | 7.92 | <0.0001 | 0.78 | 29.98 | 21.88 | 8.11 | 29.19 | <0.0001 |
| Bogota, Colombia | -1.83 | 0.0673 | -0.20 | 26.60 | 26.72 | -0.12 | 1.43 | 0.2320 |
| Cape Town, South Africa | 8.52 | <0.0001 | 1.09 | 26.29 | 23.45 | 2.84 | 10.05 | 0.0016 |
| Cebu, Philippines | -3.68 | 0.0003 | -0.25 | 19.89 | 20.65 | -0.76 | 0.11 | 0.7420 |
| Chiapas, Mexico | 1.94 | 0.0529 | 0.23 | 32.56 | 24.96 | 7.59 | 13.06 | 0.0003 |
| Crete, Greece | 0.75 | 0.4540 | 0.05 | 20.28 | 17.62 | 2.66 | 7.52 | 0.0062 |
| Fukushima, Japan | 11.75 | <0.0001 | 1.13 | 28.89 | 23.15 | 5.74 | 34.75 | <0.0001 |
| Ica, Peru | 7.93 | <0.0001 | 0.51 | 17.92 | 22.97 | -5.05 | 4.69 | 0.0307 |
| Israel | 5.15 | <0.0001 | 0.46 | 27.41 | 24.34 | 3.07 | 6.68 | 0.0099 |
| Kariba Dam, Zimbabwe | 9.39 | <0.0001 | 0.79 | 18.64 | 23.60 | -4.97 | 4.41 | 0.0361 |
| Le Reduit, Mauritius | 7.87 | <0.0001 | 0.74 | 24.20 | 16.17 | 8.03 | 50.55 | <0.0001 |
| Lujeri, Malawi | -0.23 | 0.8179 | -0.02 | 25.77 | 22.70 | 3.07 | 5.23 | 0.0225 |
| Monkey Hill, St. Kitts | 18.60 | <0.0001 | 2.29 | 30.29 | 22.74 | 7.55 | 100.58 | <0.0001 |
| Montpellier, France.139 | 14.80 | <0.0001 | 1.50 | 25.02 | 21.71 | 3.31 | 28.95 | <0.0001 |
| Montpellier, France.140 | 4.22 | <0.0001 | 0.32 | 21.16 | 18.79 | 2.37 | 3.17 | 0.0754 |
| Montpellier, France.141 | 7.64 | <0.0001 | 0.55 | 19.87 | 21.37 | -1.50 | 0.02 | 0.8803 |
| Montpellier, France.142 | 13.93 | <0.0001 | 1.69 | 31.29 | 23.13 | 8.16 | 81.37 | <0.0001 |
| Montpellier, France.143 | 3.32 | 0.0010 | 0.26 | 22.84 | 21.99 | 0.85 | 0.60 | 0.4401 |
| Montpellier, France.144 | 0.48 | 0.6305 | 0.04 | 26.61 | 22.20 | 4.41 | 0.77 | 0.3795 |
| Ogasawara Islands, Japan | 1.72 | 0.0865 | 0.19 | 25.31 | 22.02 | 3.29 | 5.83 | 0.0160 |
| Plainville, Connecticut | 8.81 | <0.0001 | 0.86 | 23.33 | 26.73 | -3.40 | 0.64 | 0.4255 |
| Pyrenees, Spain | 3.82 | 0.0001 | 0.38 | 23.77 | 25.29 | -1.52 | 0.78 | 0.3789 |
| Queensferry, Scotland | 4.98 | <0.0001 | 0.42 | 20.24 | 21.60 | -1.36 | 2.43 | 0.1198 |
| Queensland, Australia | 9.92 | <0.0001 | 1.08 | 24.99 | 27.71 | -2.72 | 2.10 | 0.1475 |
| San Luis Potosi, Mexico | 8.01 | <0.0001 | 0.80 | 25.72 | 21.02 | 4.70 | 38.20 | <0.0001 |
| Southwest Harbor, Maine | 3.30 | 0.0010 | 0.31 | 20.72 | 22.32 | -1.59 | 1.96 | 0.1616 |
| Sussex, United Kingdom.161 | 7.51 | <0.0001 | 0.46 | 22.54 | 14.35 | 8.19 | 44.75 | <0.0001 |
| Sussex, United Kingdom.162 | 5.04 | <0.0001 | 0.35 | 17.68 | 22.37 | -4.70 | 0.40 | 0.5261 |
| Tananarive, Madagascar | 10.03 | <0.0001 | 0.90 | 24.07 | 22.18 | 1.89 | 1.75 | 0.1861 |
| **Cross strain** |  |  |  |  |  |  | **t-test:  t = 2.755** | |
| **Average** ***CV_E_*** |  |  |  | **24.07** | **22.05** |  |  | **p = 0.0072** |

(Δmean = male mean -female mean and Δ*CV_E_* =male *CV_E_* -female *CV_E_*)

# APPENDIX 3
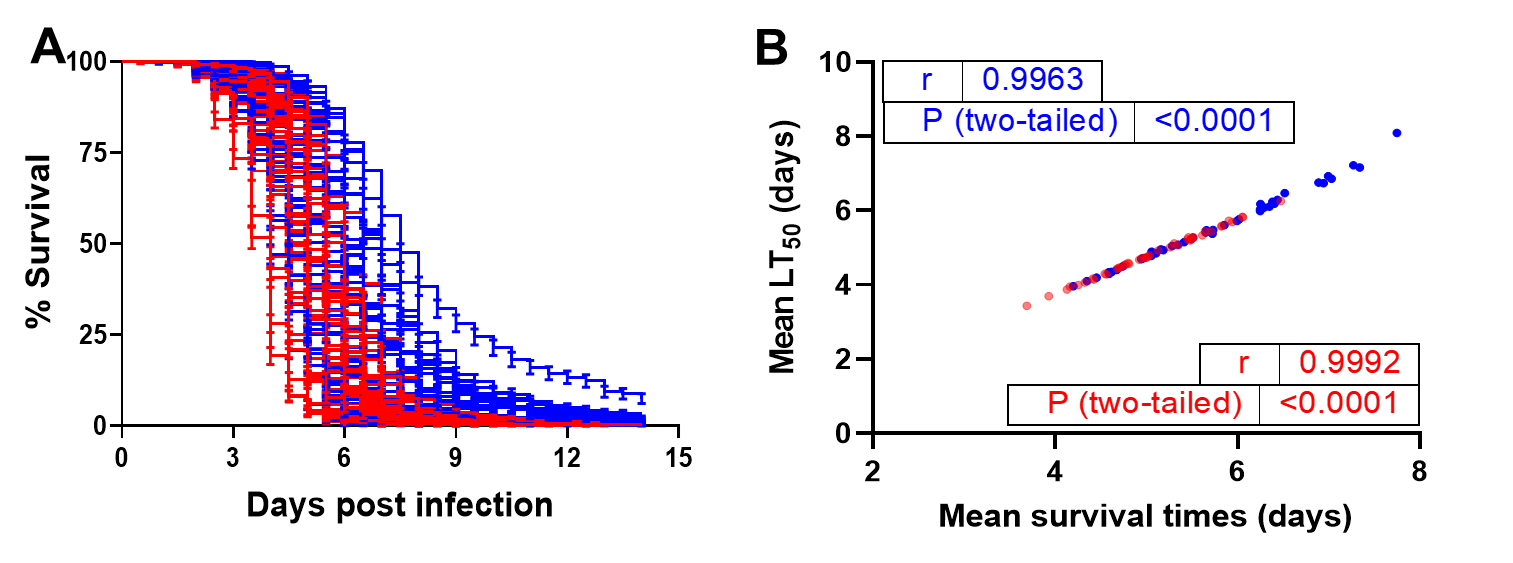

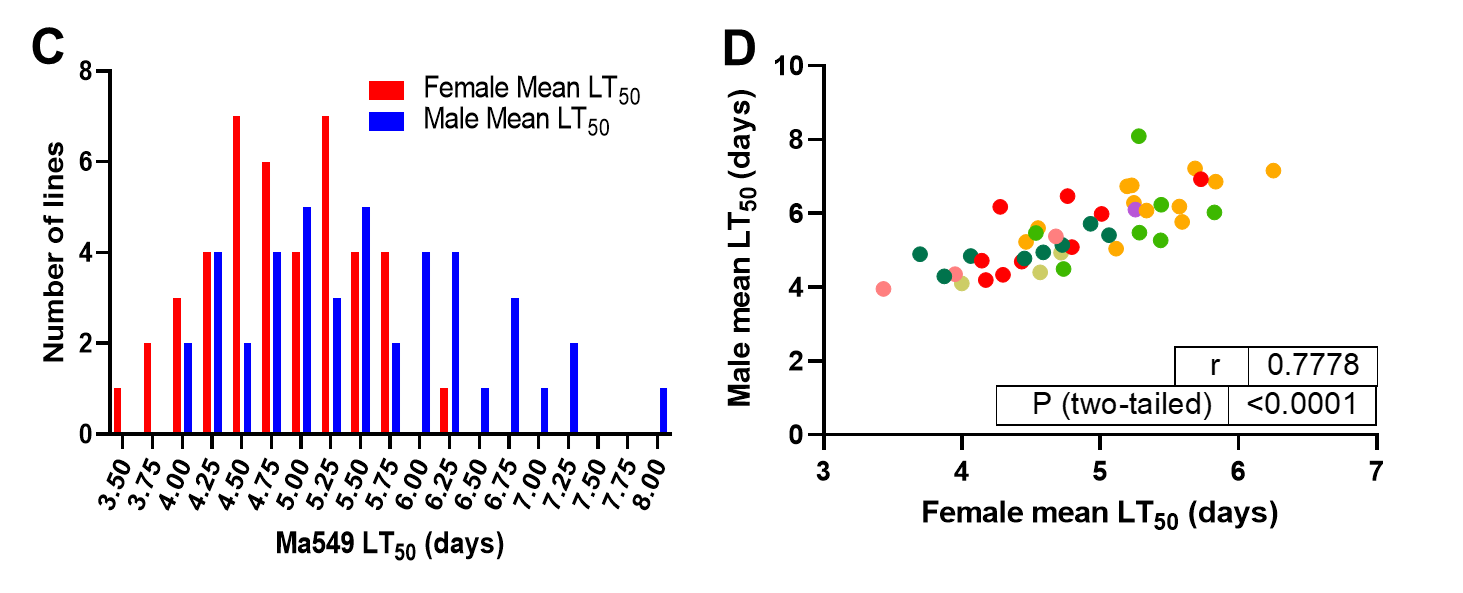


**APPENDIX** 3 **(A)** The survival data of 43 *Drosophila* populations infected with Ma549. The mean percentage survival ± SEM for females (red, n = 281-501) and males (blue, n = 279-497) across 10-15 replicates per sex per line in 2-3 experiments. **(B)** The correlation between mean survival times and LT_50_ values in male (blue dots) and female (red dots) lines infected with Ma549, with Pearson correlation coefficient (r) and p-values indicated. **(C)** The distribution of longevity in females (red bars) and males (blue bars) confirming the normal distribution of mean LT_50_ values from the 43 lines using D'Agostino & Pearson and Shapiro-Wilk tests (p > 0.05). **(D)** Correlation analysis of LT_50_ values between the sexes, including the Pearson correlation coefficient (r) and p-values.

# APPENDIX 4


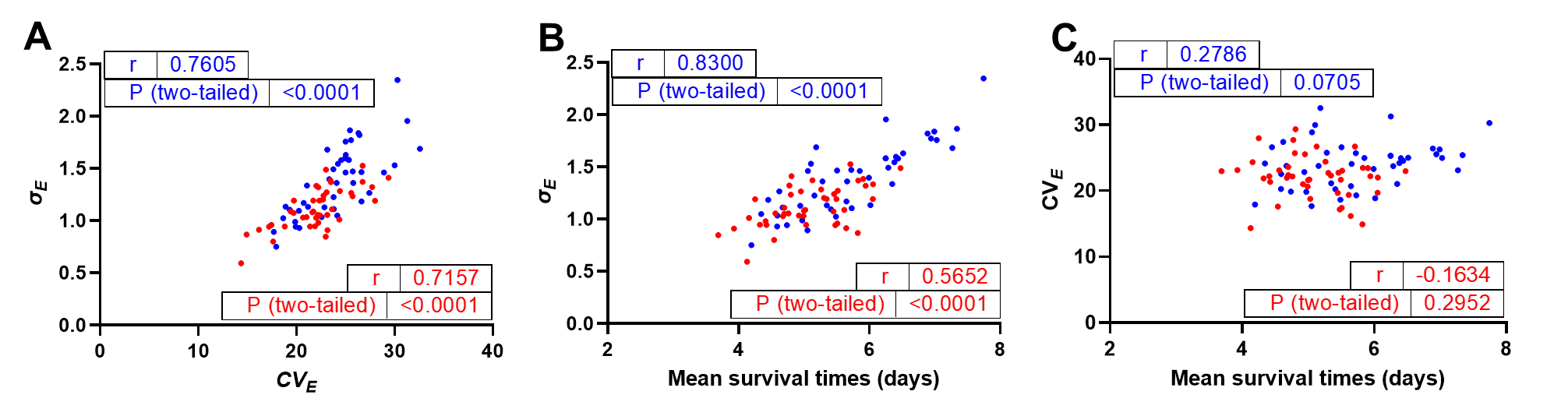

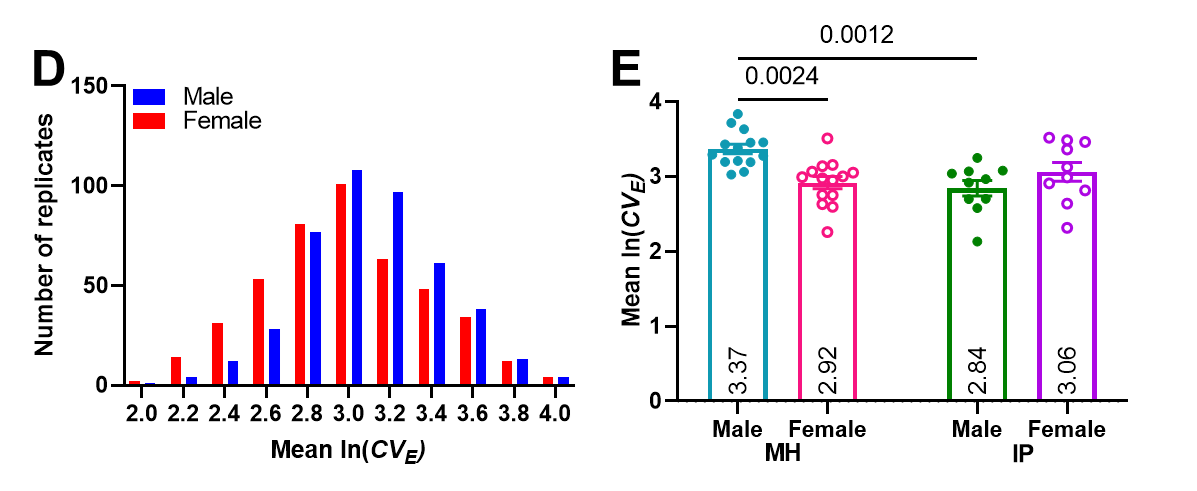

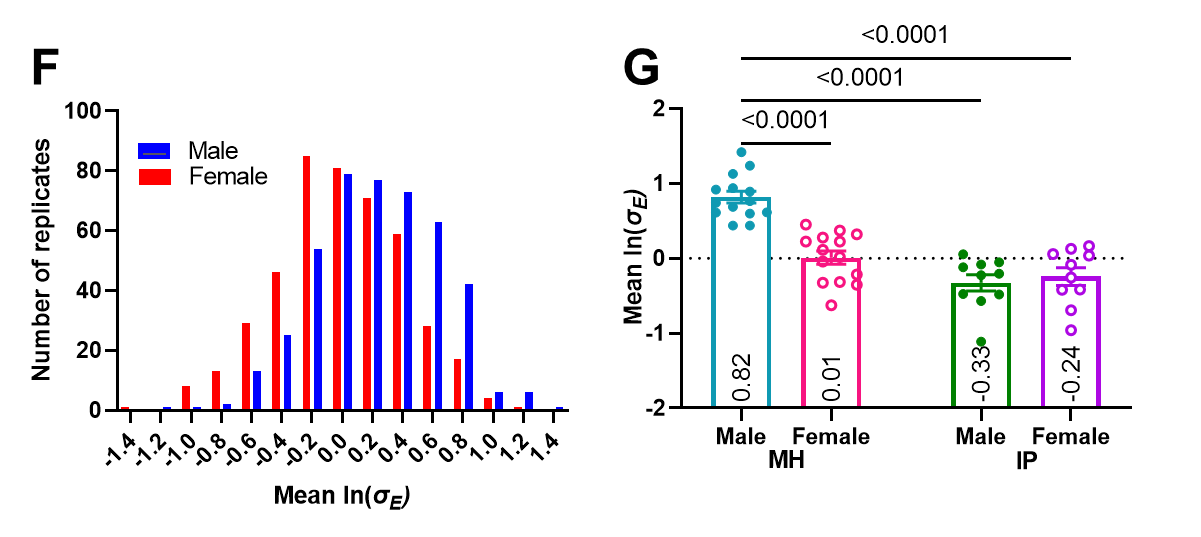
**APPENDIX 4** Correlation analyses of plasticity in global lines exposed to Ma549 are presented for male and female *σ_E_* versus *CV_E_* **(A)**, *σ_E_* versus MST **(B)**, and *CV_E_* versus MST **(C)**, with corresponding r and p-values. Blue and red dots in these figures represent males and females from all 43 lines, respectively. Plasticity distribution **(D, F)** and bar graph **(E, G)** of ln(*CV_E_*) **(D, E)** and ln(*σ_E_*) **(F, G).** 443 global male (blue) and female (red) replicates are represented in panels D and F, while 14 replicates with 373 MH males (turquoise) and 501 females (crimson) from three trials, 10 replicates with 344 IP males (green) and 281 females (purple) from two trials are depicted in panels E and G. The transformed ln(*σ_E_*) and ln(*CV_E_*) show a normal distribution in D and F. Panels E and G use two-way ANOVA (Tukey's multiple comparisons) to analyze MH and IP plasticity, presenting mean ± SEM and p-values.

# APPENDIX 5

**Geographic variables impact host susceptibility to infection**

Two-way ANOVA demonstrated significant main effects on fly longevity, with sex accounting for 5.549% (F_(1, 456)_ = 29.37, p < 0.0001), and geographical origins for 8.296% (F_(4, 456)_ = 10.98, p < 0.0001) of the total variation (Appendix 1: Table A8B). DGRP males (females) were 1.09 (1.10)-fold more resistant than European/Middle Eastern males (females) (p = 0.0207).

**APPENDIX 5** Geographic variation in LT_50_ values among worldwide fly lines infected with Ma549. The 43 worldwide fly lines are depicted with dots: pink for African, green for Europe/Middle East, blue for Asian/Pacific, and red for American regions. LT_50_ values were averaged for each of the 43 male and female lines, with data obtained from 10-15 replicates (averaging 35 flies each) per sex per line across 2-3 experiments. Data from previously published DGRP lines are shown in black(Wang et al., 2017). A two-way ANOVA was conducted to evaluate the main effects of geographic region and sex on disease resistance. The analysis revealed no significant interaction between region and sex (F_(4, 456)_ = 2.261, p = 0.0617). Least squares means (LSM) ± SEM from Tukey's multiple comparison test are shown, with a compact letter display (CLD) positioned at the top of individual bars, with statistical significance where bars are not connected by the same letter.

# APPENDIX 6

**Linear regression analyses of latitude, altitude, and climate variables for global fly collection sites (2005-2010)**

Using NASA Power Data, we analyzed hourly temperature and relative humidity from 2005 to 2010 at 28 collection sites, corresponding to the collection dates of most of the 43 fly lines (Appendix 1: Table A3). Using NASA Power Data to analyze hourly temperature, we are able to detect very short interludes with extreme conditions, such as a reading of 43.46°C in Australia. We recorded mean annual hourly minimum, maximum, and range (maximum - minimum) values. Linear regressions show strong correlations between collection site latitude (Table 1) and minimum annual temperature (Tmin) (A) and annual temperature range (Trange) (C), but not maximum annual temperature (Tmax) (B). Altitude (Table 1) moderately correlates with minimum annual relative humidity (RHmin) (D) and annual relative humidity range (RHrange) (F), but not maximum annual relative humidity (RHmax) (E). The narrow ranges of maximum annual temperature and humidity explain the lack of correlations with latitude or altitude.


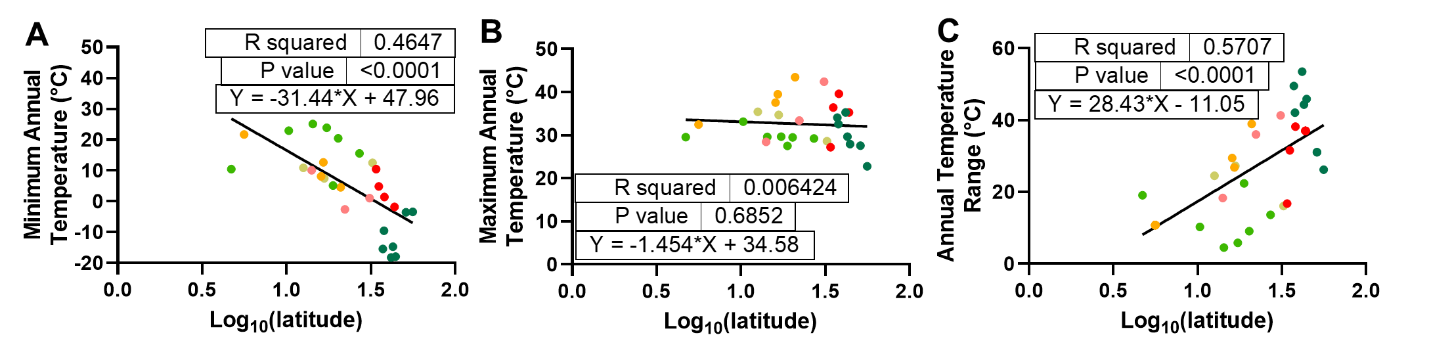

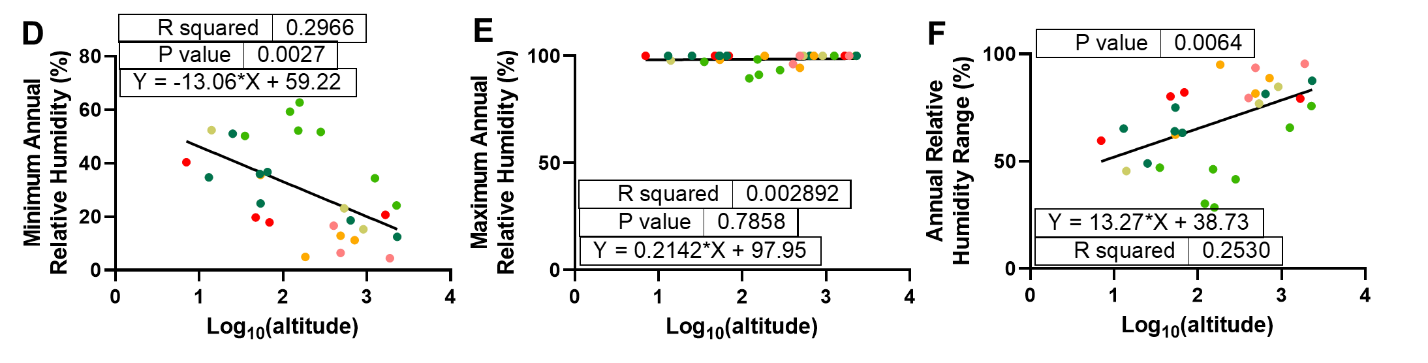
**APPENDIX 6** Simple linear regression analyses between latitude and annual temperatures or altitude and relative humidities for the global fly population collection sites: Biome colors across 28 geographic locations (2005-2010) match those in Figure 2A.The figures show results for minimum annual temperature **(A)**, maximum annual temperature **(B)**, annual temperature range **(C)**, minimum annual relative humidity **(D)**, maximum annual relative humidity **(E)**, and annual relative humidity range **(F)**, including R^2^ values, p-values, and regression equations with slopes.

# APPENDIX 7

**Simple linear regression analyses correlating Ma549 LT_50_ values with latitude, altitude, annual temperatures or precipitations at collection sites for global fly populations**

**
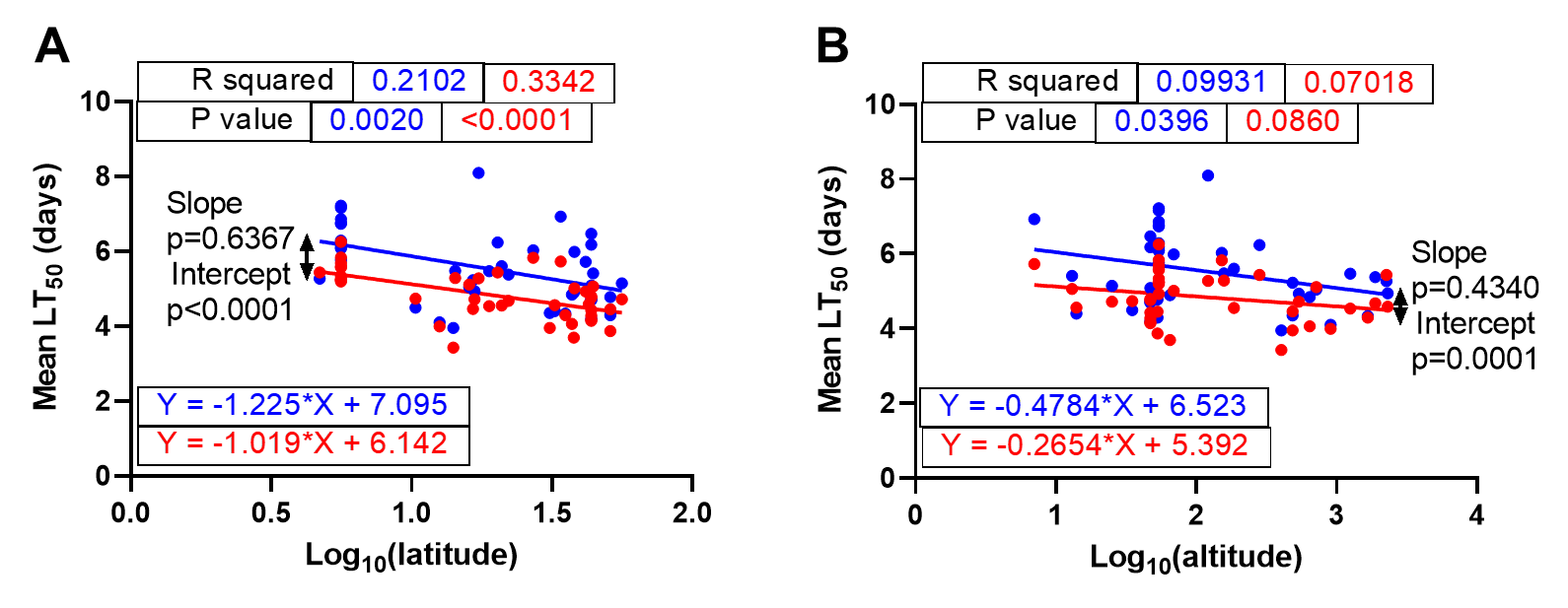
**


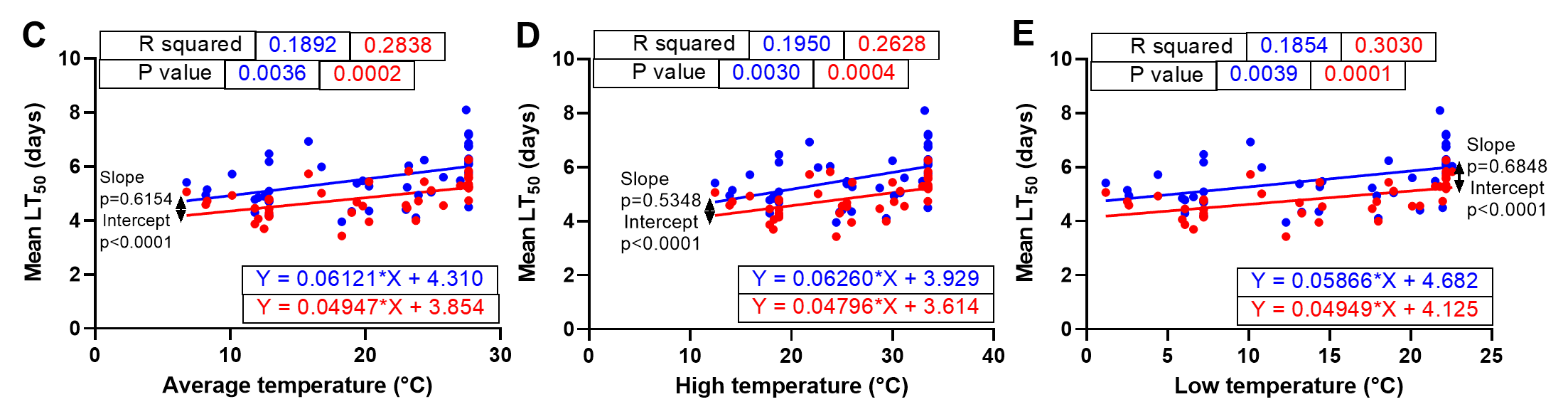

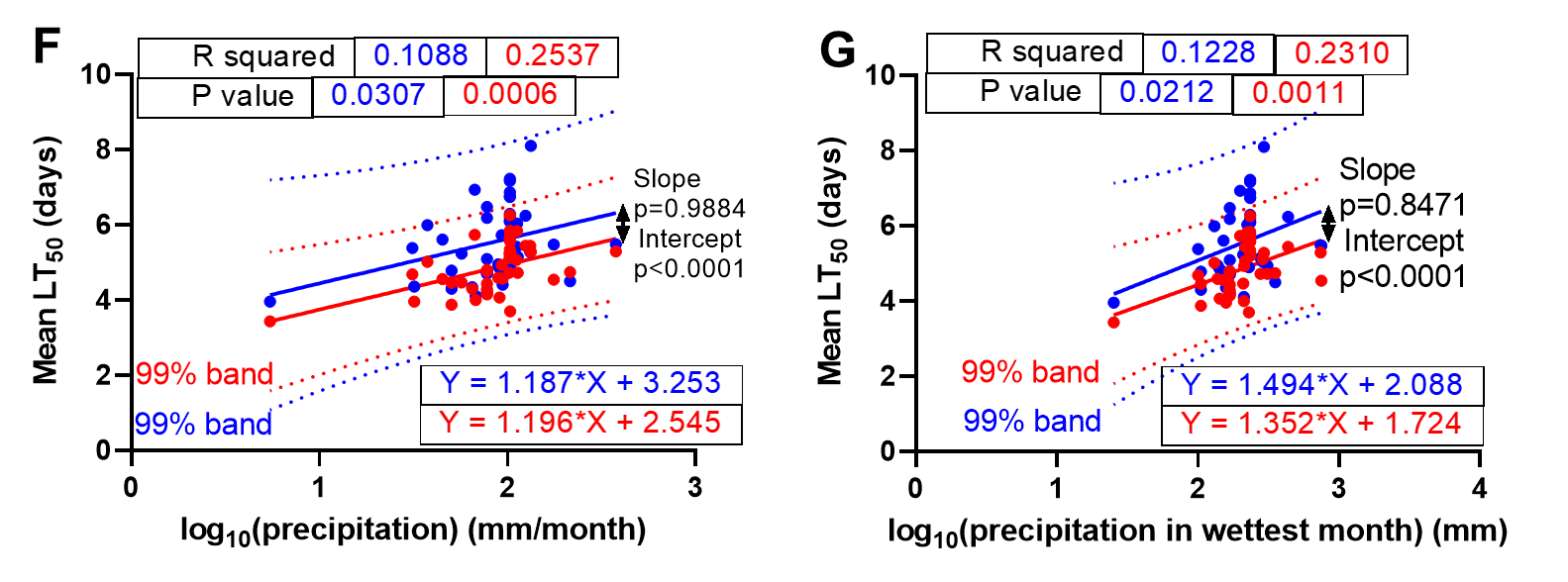
**APPENDIX 7** Simple linear regression analyses correlating Ma549 LT_50_ values with latitude, altitude, annual temperatures or precipitations at collection sites for global fly populations: Temperature and precipitation data were extracted from all 28 collection sites based on the original collection year for each fly line. Results for latitude (**A**), altitude (**B**), annual average temperatures (**C**), high temperature (**D**), low temperature (**E**), annual mean precipitation (**F**), and precipitation in wettest month (**G**) are shown, featuring R^2^ values, p-values, and regression equations including slopes. Male (female) regression lines are in blue (red), with p-values for comparing slopes and intercepts between female and male lines using analysis of covariance (ANCOVA).

# APPENDIX 8

**The effects of annual temperature and relative humidity on female and male LT_50_ and plasticity values**

To investigate disease resistance, we calculated the average annual exposure to the preferred temperature (20-30°C) using 52,584 hourly data points from 2005 to 2010 (Appendix 1: Table A3A). Exposure at collection sites ranged from 75 to 8,764 hours/year (A-C).

Linear regression analyses for the 43 fly populations showed strong correlations between mean survival time (MST) in response to Ma549 for males and females and experiencing longer aseasonal uniformity measured as the hours per year spent within preferred temperature ranges (A). Positive correlations were also found between *σ_E_* and increasing aseasonal uniformity in males, but not females (B). We observed no evidence of correlation between *CV_E_* values and increasing aseasonal uniformity in males and females (C). Despite differing intercepts due to males' greater resistance, the MST values for both sexes trended similarly with temperature, resulting in nearly parallel regression slopes. Higher R² values for females suggest that temperature have a greater impact on female disease resistance.


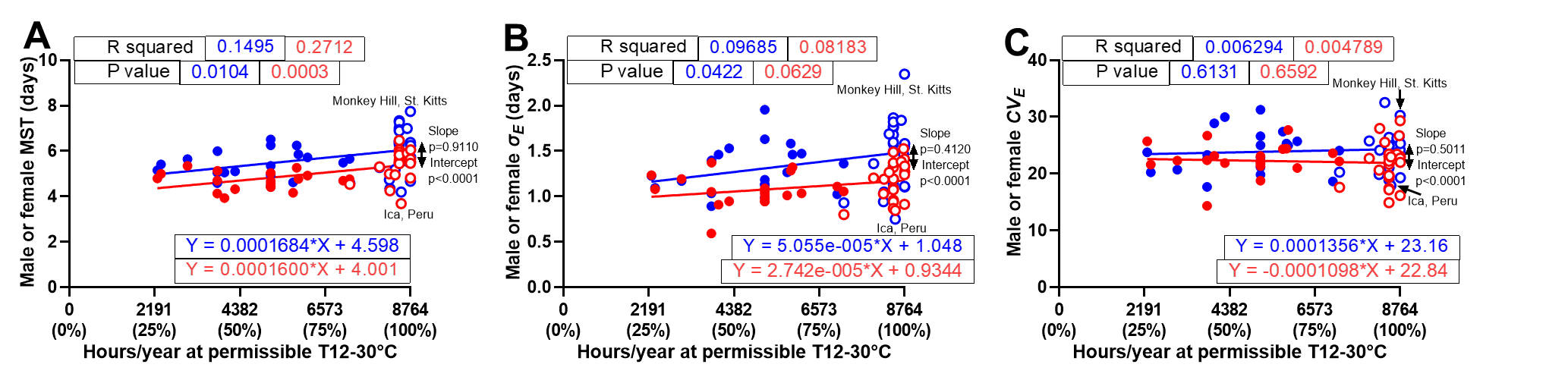

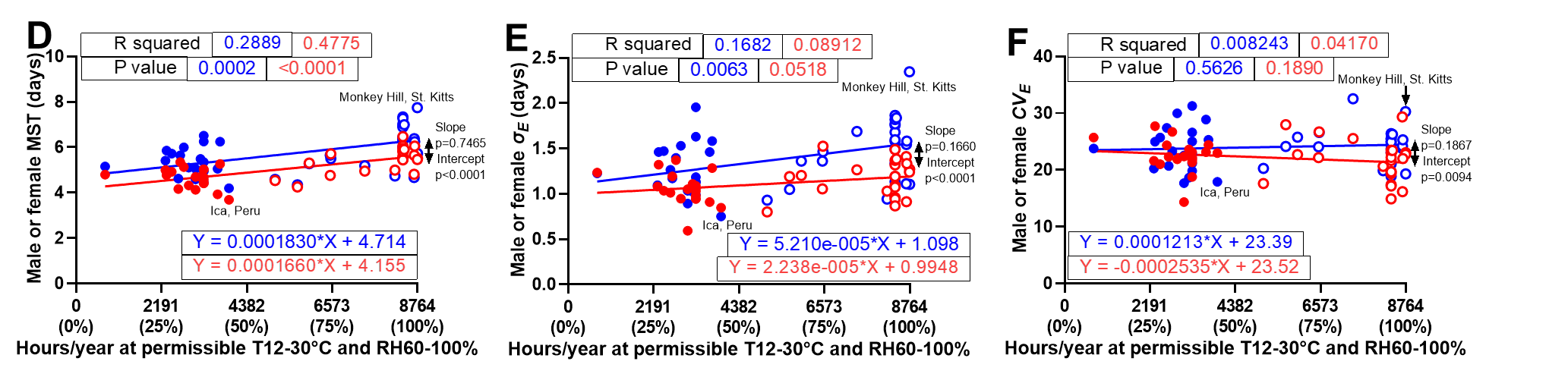
**
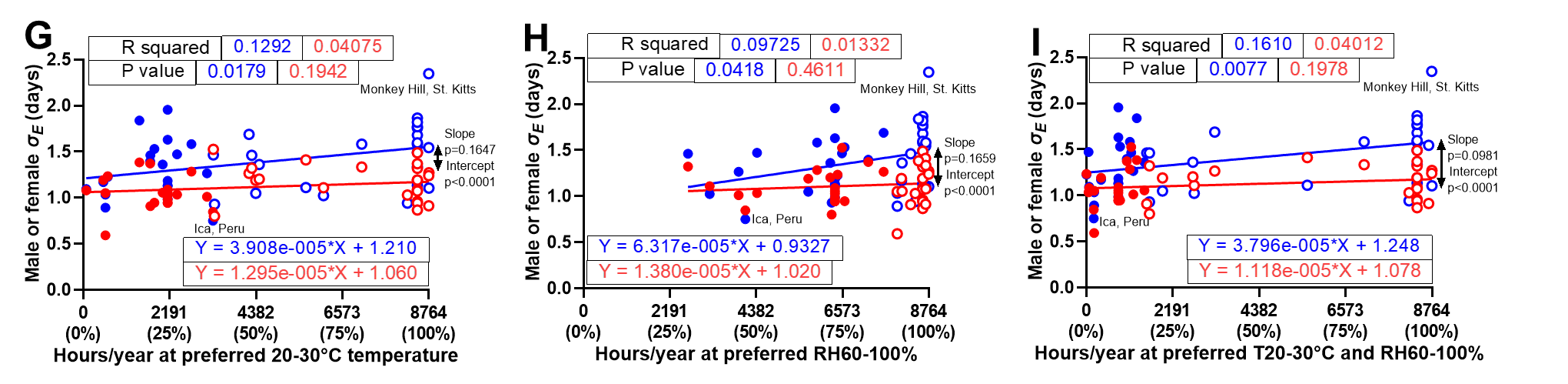
APPENDIX 8** Simple linear regression analyses correlating Ma549 MST, *σ_E_*, and *CV_E_* values with hours of permissible temperature (12-30°C) with and without relative humidity (60-100%) or *σ_E_* values with preferred temperature (20-30°C), or preferred relative humidity (60-100%) or preferred temperature (20-30°C) coinciding with preferred relative humidity (60-100%) at collection sites for global fly populations. The analysis uses 52,584 hourly data points from 2005-2010 from all 28 sites for each fly line (Appendix 1: Table A3A)**.** Results are shown for permissible temperature with MST (**A**), *σ_E_* (**B**), and *CV_E_* (**C**), and for permissible temperature coinciding with relative humidity with MST (**D**), *σ_E_* (**E)**, and *CV_E_* (**F**), as well as *σ_E_* values with preferred temperature (**G**), or preferred relative humidity (**H**) or preferred temperature coinciding with preferred relative humidity (**I**) at collection sites for global fly populations, including R^2^ values, p-values, and regression equations with slopes. Male (female) regression lines are in blue (red). Male and female regression lines are blue and red, respectively. Aseasonal flies are depicted as circles, and seasonal flies as solid dots; this distinction applies to all subsequent figures. Males exhibited a positive correlation between *σ_E_* plasticity and exposure to permissible or preferred conditions (B, E, G-I).

# APPENDIX 9


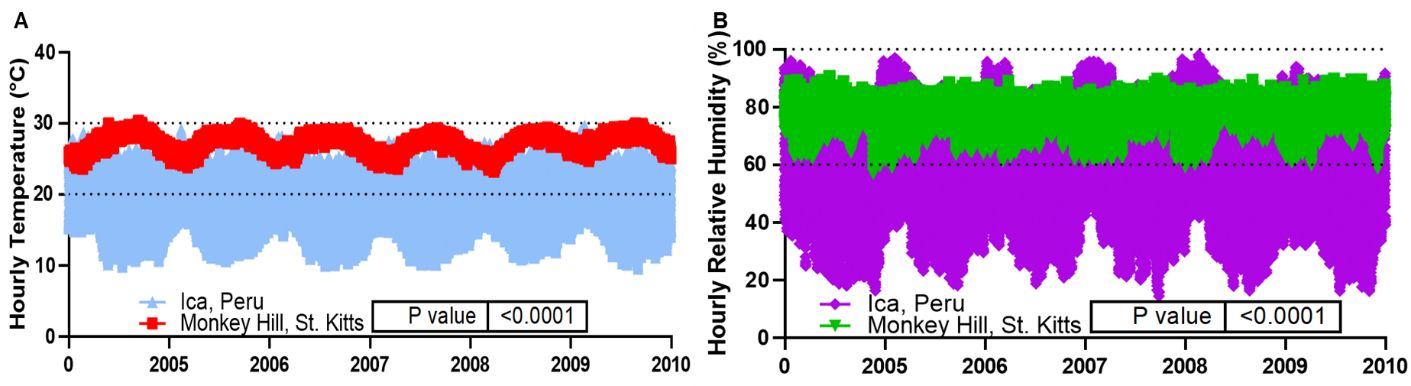

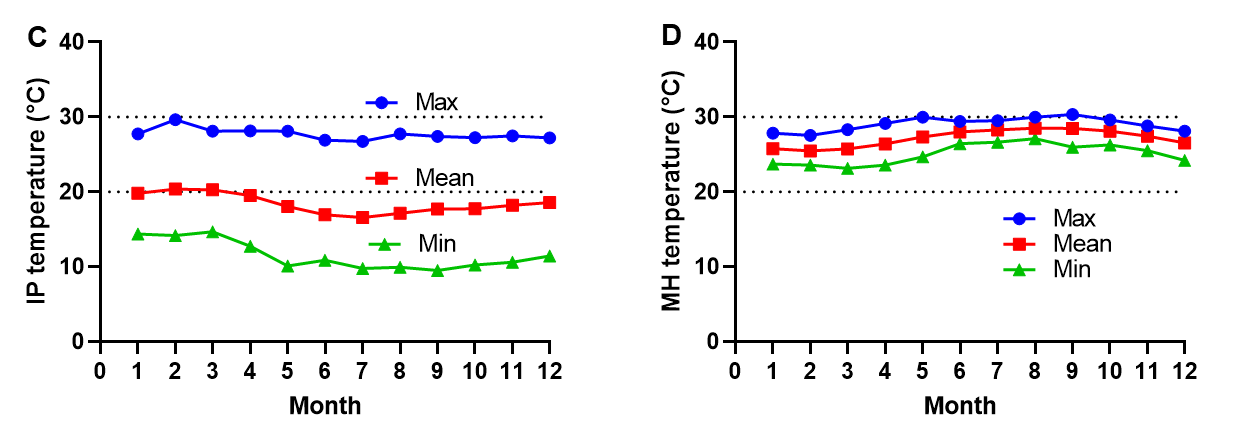

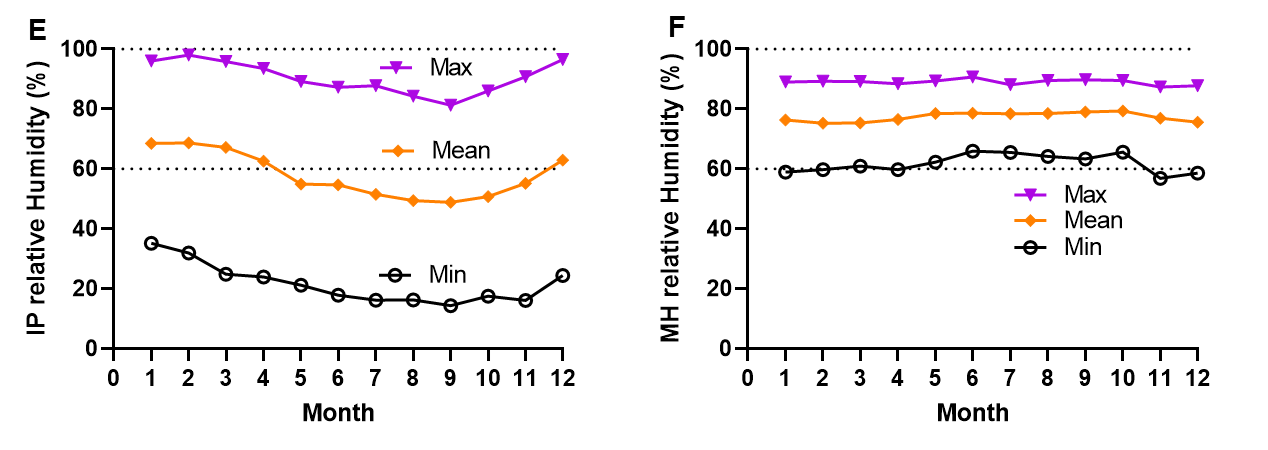

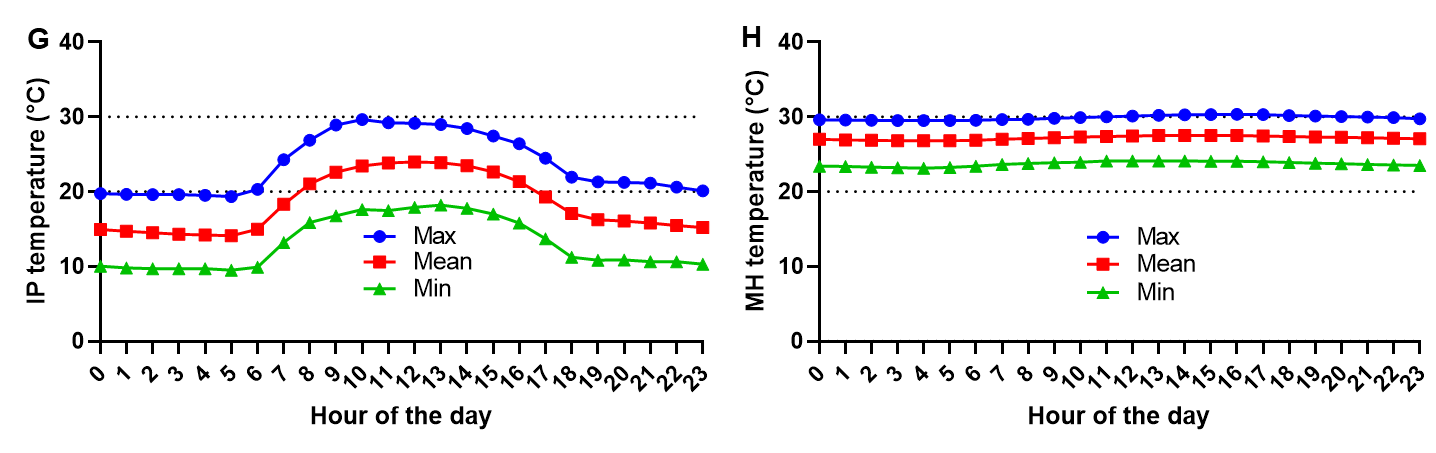

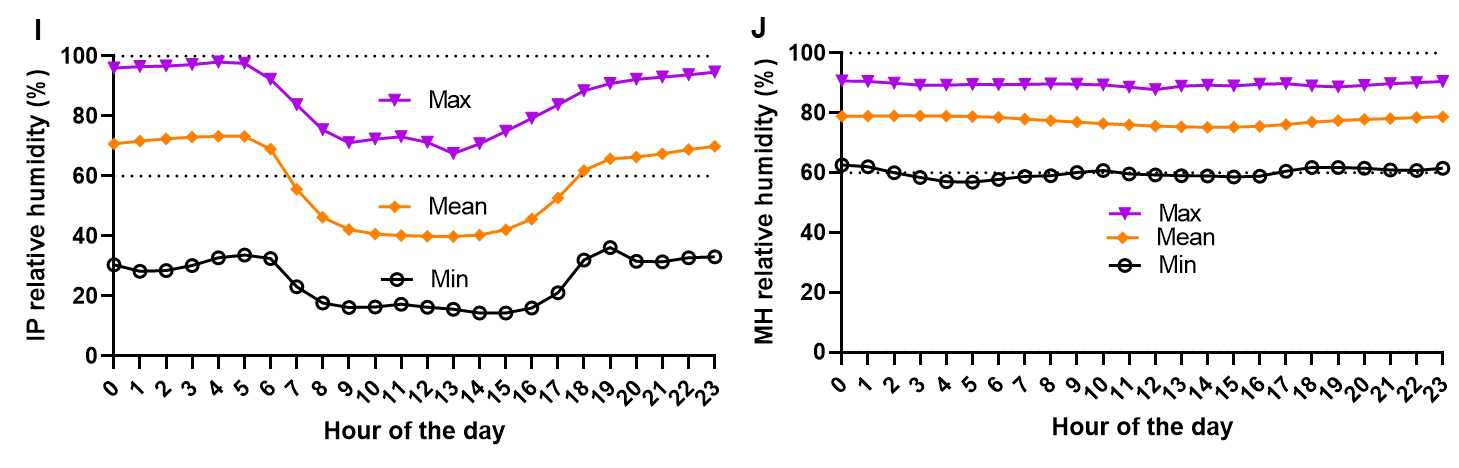
**APPENDIX 9** Hourly temperature **(A)** and relative humidity **(B)** data for Monkey Hills, St. Kitts (MH) and Ica, Peru (IP) are shown through Lomb-Scargle periodograms, capturing hourly fluctuations from January 01, 2005, to December 31, 2010 (n=52,584). Comparisons were made using Welch’s t-test with mean ± SEM and p-values indicated. Monthly temperature **(C, D)** and relative humidity **(E, F)** plots for IP **(C, E)** and MH **(D, F)** display maximum, mean, and minimum values across months from 2005-2010 (n=52,584 hourly data points), identifying seasonal trends and temperature-humidity interactions. Hourly temperatures (G, H) and relative humidity **(I, J)** plots for IP **(G, I)** and MH **(H, J)** illustrate daily patterns of maximum, mean, and minimum temperatures and relative humidity from 2005-2010 (n=52,584 hourly data points). These visuals help identify seasonal variations and trends in temperature and humidity interactions.

# APPENDIX 10

**Effects of line, sex, mating status, and infection on sleep**

ANOVA was used to validate the effects of genetic line, sex, mating status, and infection on nighttime (Appendix 11A) and daytime (Appendix 11B) sleep, as detailed in Table 1 (derived from the data in Appendix 1: Table A5B). The analysis confirmed that infection status significantly affected daytime sleep (p < 0.0001) but not nighttime sleep (p = 0.5364), highlighting infection’s role in altering sleep at different times. Additionally, mating status had a much stronger impact on daytime sleep. A significant three-way interaction among line, infection, and sex (p ≤ 0.0020) was observed for both sleep periods, indicating that the influence of one factor on sleep can be significantly modified by the others.

**APPENDIX 10** Effects of line, sex, mating status, and infection as well as their interactions on night and daytime sleep

| **Source** | **Nparm** | **DF** | **Nighttime sleep** | | | **Daytime sleep** | | |
| --- | --- | --- | --- | --- | --- | --- | --- | --- |
|  |  |  | **Sum of Squares** | **F Ratio** | **p value** | **Sum of Squares** | **F Ratio** | **p value** |
| line | 2 | 2 | 4286291.9 | 874.5660 | <0.0001 | 1876451.8 | 219.7682 | <0.0001 |
| Infection | 1 | 1 | 937.4 | 0.3825 | 0.5364 | 3622098.1 | 848.4331 | <0.0001 |
| Mating | 1 | 1 | 17285.3 | 7.0537 | 0.0081 | 422367.3 | 98.9345 | <0.0001 |
| Sex | 1 | 1 | 1024007.7 | 417.8727 | <0.0001 | 17504.2 | 4.1001 | 0.0432 |
| line*Infection | 2 | 2 | 4138.2 | 0.8444 | 0.4302 | 34266.2 | 4.0132 | 0.0185 |
| line*Mating | 2 | 2 | 8143.3 | 1.6615 | 0.1906 | 2587.0 | 0.3030 | 0.7387 |
| line*Sex | 2 | 2 | 1519311.5 | 309.9971 | <0.0001 | 73329.8 | 8.5883 | 0.0002 |
| Infection*Mating | 1 | 1 | 2616.3 | 1.0676 | 0.3018 | 7005.3 | 1.6409 | 0.2006 |
| Infection*Sex | 1 | 1 | 132418.2 | 54.0366 | <0.0001 | 915803.3 | 214.5160 | <0.0001 |
| Mating*Sex | 1 | 1 | 694.8 | 0.2835 | 0.5945 | 22777.7 | 5.3354 | 0.0212 |
| line*Infection*Mating | 2 | 2 | 90.1 | 0.0184 | 0.9818 | 21402.3 | 2.5066 | 0.0822 |
| line*Infection*Sex | 2 | 2 | 30723.9 | 6.2688 | 0.0020 | 99378.2 | 11.6391 | <0.0001 |
| line*Mating*Sex | 2 | 2 | 1820.1 | 0.3714 | 0.6899 | 15843.5 | 1.8556 | 0.1571 |
| Infection*Mating*Sex | 1 | 1 | 1845.4 | 0.7531 | 0.3858 | 162.7 | 0.0381 | 0.8453 |
| line*Infection*Mating*Sex | 2 | 2 | 6.5 | 0.0013 | 0.9987 | 15603.2 | 1.8274 | 0.1615 |

(Source: This is a list of the tested factors and interactions. Nparm: Number of parameters associated with the effect. DF: degrees of freedom for each source of variation. Sum of Squares: sum of squares (SS) for each source of variation, along with the total from all sources. F Ratio: the mean square of the factor (lot) divided by the mean square of the error. A p-value less than 0.05 means that the factor or interaction has a statistically significant effect.)

# APPENDIX 11


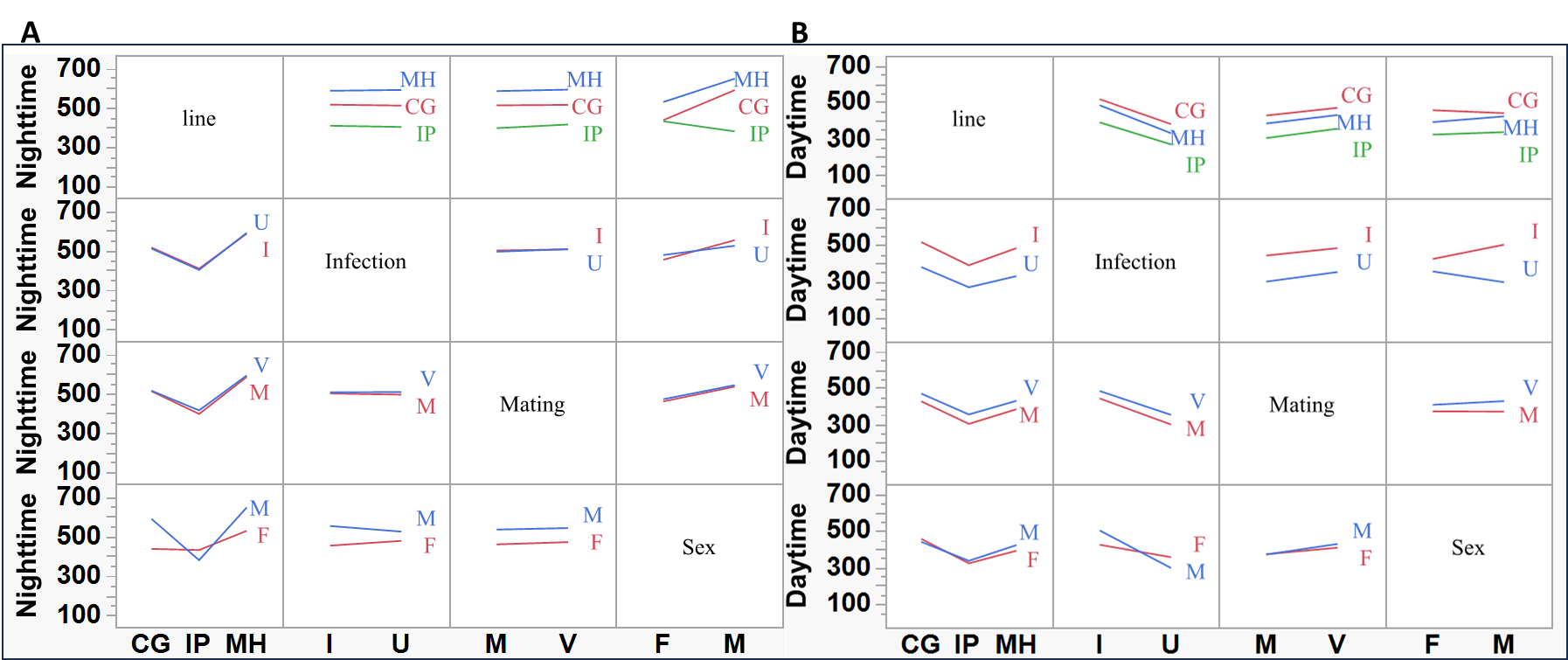


**APPENDIX 11** Interaction profiles of line, sex, mating status, and infection status on nighttime **(A)** and daytime **(B)** sleep (min/12h) across MH, CG, and IP females and males. These profiles were derived from the sleep data of 768 individual flies, considering three genetic lines (MH, CG, IP), both sexes (females and males), two mating statuses (mated and virgin), and two infection statuses (infected and uninfected). Abbreviations: sex (F = female, M = male), mating status (M = mated, V = virgin), and infection status (I = infected, U = uninfected).
